# A novel hybrid clustering approach for robust ramp event characterization

**DOI:** 10.1038/s41598-026-51147-0

**Published:** 2026-06-20

**Authors:** M. Saber Eltohamy

**Affiliations:** Department of Power Electronics and Energy Conversion, Electronics Research Institute, Cairo, 12622 Egypt

**Keywords:** Renewable generation, Wind variability, Power ramps categorization, k-means clustering, Standard deviation scores (Z-scores), DBSCAN, Energy science and technology, Engineering, Mathematics and computing

## Abstract

Large power fluctuations in a brief amount of time, or ramp events, are an increasing concern for grid operators due to the rise in renewable energy generation and the unreliable hour-ahead predictions. To balance these ramp events, grid operators need to be aware of their anticipated occurrence intervals and range. Prior studies used binary ramp event categorization, whereas other studies employed non-causative classification techniques. Existing clustering methods, Z-score and k-means, have strengths but distinct limitations. To address these, this paper introduces the ZK-means hybrid approach, integrating Z-score normalization with k-means partitioning, forming a centroid-based clustering algorithm to enhance adaptability, noise resistance, and interpretability in ramp classification. Its need arises from the growing demand for accurate and efficient ramp analysis to support reliable grid operation and forecasting. Two comparison phases for the ZK-means approach were conducted: First, it was evaluated against its constituent methods to assess the benefits of their combination; second, it was compared to the density-based spatial clustering of applications with noise (DBSCAN) algorithm to verify its robustness and general applicability. Although DBSCAN can capture local variations in data, it produced inconsistent cluster numbers and required frequent parameter tuning across the ten years. In contrast, ZK-means achieved more stable clustering patterns and lower within-cluster variance, demonstrating superior reliability for long-term ramp event characterization. The new categorization method is applied to a real case study, and the results reveal that the new hybrid method offers significant improvements in the quality, robustness, and interpretability of the clustering process and its resulting cluster characteristics, as it combines the stability of normalization with the scalability of k-means, offering a robust and practical solution for large-scale, high-dimensional clustering. While this new method does entail a slight increase in time-speed, computational complexity, and energy consumption compared to its constituent methods, it remains faster than DBSCAN and the enhanced insights it provides offer critical advantages for effective grid management.

## Motivation

Ramp events in variable renewable generation (VRG), mainly from wind and solar sources, represent significant challenges to the power system operator, affecting grid stability, demand balancing, and forecasting accuracy. Traditional clustering methods, such as Z-score-based range classification and k-means clustering, each offer dissimilar advantages but also have inherent limitations. The Z-score clustering technique provides clear statistical interpretation and sensitivity to deviations, but needs adaptability in capturing spatial patterns across ramp magnitudes. k-means efficiently partitions datasets into compact groups, but it is sensitive to outliers and scale variations in ramp data. These constraints underscore the necessity for a hybrid clustering framework capable of integrating statistical normalization with centroid-based optimization. Therefore, this paper proposes the ZK-means clustering approach that combines the robustness of Z-score normalization with the partitioning efficiency of k-means, assuring consistent scaling, noise resistance, and interpretable power ramp classification, and verifying its robustness against the DBSCAN algorithm. The motivation derives from the growing need for accurate, adaptable, and computationally efficient clustering approaches to enhance renewable power ramp analysis, facilitate grid operation decisions, and support the development of forecasting systems.

## Introduction

Wind and solar energy, as types of VRG, are gaining significance in the global energy portfolio. Nonetheless, the intrinsic variability and uncertainty associated with these energy sources pose significant challenges for power system operators ^1,2^. Traditional approaches to power grid management frequently encounter challenges in addressing the irregular and inconsistent production of renewable energy, leading to inefficiencies and potential reliability issues. To address these issues, research focuses on two main areas: developing grid-side and demand-side flexibility through the coordinated use of dynamic thermal line rating and electric vehicle scheduling ^3–11^, and implementing advanced data-driven models, such as hybrid clustering and ensemble machine learning approaches, for the accurate characterization of ramp events and multi-timescale forecasting ^12–16^. In ^17^, two wind forecast systems were evaluated, and both systems exhibit reduced predictive capability due to their tendency to underestimate strong winds and overestimate weak winds. Nevertheless, the models were capable of accurately representing generation events that last between 5 and 10 h. The study illustrated the importance of evaluating operational models concerning wind power ramps and persistence events to improve regional wind forecasting for efficient wind resource management. The temporal characteristics of various electrical variables in power systems, such as voltage, current, and power, highlight the need to employ specialized clustering techniques that are tailored for time-series data. These specialized algorithms are capable of capturing the temporal relationships and patterns that are inherent in power system data. Data preprocessing methods can improve power ramp categorization, as advanced data-driven techniques, such as k-means clustering, have emerged as promising solutions for optimizing the integration of VRG into power systems. Due to the growing availability of data and advances in computational power, clustering algorithms such as k-means are now indispensable tools to analyze, optimize, and modernize power systems ^18^, where clustering denotes the process of partitioning data into discrete groups comprising similar items. The recent publications on modern power systems have exhibited a marked increase in the application of clustering algorithms, demonstrating a significant upward trend, as clustering algorithms are vital for organizing large datasets into coherent and significant clusters, uncovering hidden patterns and structures. This facilitates automated decision-making driven by data, managing a substantial volume of data points. They assist in formulating power management strategies, enhancing overall operational efficiency and reliability ^19^. The k-means algorithm is commonly used for clustering analysis in power systems. This is due to its effectiveness, versatility in handling different forms of data, ability to handle large datasets, and simplicity of implementation. Consequently, it is frequently selected as a benchmark for evaluating the effectiveness of newer or lesser-known algorithms ^19,20^. The algorithm is iterative and involves dividing a dataset into *k* non-overlapping subgroups or clusters. It is a highly suitable option for various applications. The findings in ^21^ demonstrate that ensemble models with clustering effectively utilize the inherent information in wind data to model wind power in a wind farm using multiple meteorological factors. As a result, these models outperform models without clustering by an average of approximately 15% in wind power modeling. Wind power ramp analysis and classification are crucial for managing power quality and reliability while lowering the cost of providing spinning reserves ^22–26^. System operators manage small ramps through generation control mechanisms, while large events are mitigated through re-dispatching or load shedding. Identifying ramp events in wind generation data is challenging due to no proven criteria ^27^. A method for classifying power ramps is needed, allowing operators to understand the probability of each level’s occurrence.

The physical method models the weather at the wind farm’s site using in-depth topological and meteorological information. For a wind farm with limited historical data, a specific wind power forecasting model may be established by the physical method. To create an accurate model, this method specifically requires knowing the physical features of wind farms and wind turbines in detail. Due to the unique geographical circumstances and wind turbine operating parameters, the model’s adaptability is limited. The statistical method seeks to determine the link between wind power and a number of variables, such as past data and measured data, to forecast wind power based on the continuity concept. The method works in the majority of situations without taking geographic factors into account.

Ramp event classification offers a powerful tool for analyzing and managing renewable generation data, enabling better decision-making and optimization of power system operations. In the binary ramp event classification, a ramp event occurs when the power variation during a specific time interval surpasses a predetermined threshold value. This value indicates the level of change that is challenging to handle within a specific timeframe. In previous studies, various threshold ramp values were used, as a result, the total number of ramp events detected was greatly influenced by the chosen threshold value, which made it challenging to compare findings from various analyses ^28,29^, with the threshold values considered ranging from 1% to exceed half of the installed renewable power, and the time interval considered varying from minutes to several hours. Moreover, ramps categorized as ramp events exhibited similarities; however, in actuality, ramp events possess distinct characteristics and necessitate the implementation of diverse operational strategies to address them. Some studies utilized a non-causative classification to categorize ramp events into multiple levels ^30–32^. Therefore, a unified framework is necessary to address the complexity arising from the heterogeneity in the classification of ramp events. Recent studies utilized the highest ramp value and standard deviation scores (Z-scores) ^22^. Classification methods should effectively differentiate ramps based on their distinct characteristics, consider the difference between power system utilities, and possess the ability to be applied universally across all power systems. Although the k-means algorithm is extensively utilized and valued for its simplicity and efficiency, it frequently fails to satisfy the nuanced demands of intricate domains such as power systems ^19^. Although k-means clustering was used with wind power data, it was not specifically applied to ramp event classification. In ^33^, three algorithms from two distinct unsupervised techniques were examined for clustering wind power patterns. Additionally, eight validity indices were utilized. The optimal pairing of clustering algorithm and validity index was determined to be k-means and Silhouette index, respectively. In ^34^, clustering methodologies have been used to reduce spatio-temporal wind speed data into statistically representative classes of temporal profiles for subsequent processing and interpretation. In ^35^, k-means clustering is used for short-term wind power forecasting to categorize the samples into multiple groups based on the similarity between historical days. These groups contain meteorological conditions and historical power data. The results demonstrated that it can achieve superior forecasting accuracy compared to other baselines and existing short-term wind power forecasting approaches. The conclusions of ^36^ indicated that employing machine learning models to optimize wind energy production is both viable and essential for enhancing the efficiency of this industry. Unsupervised models, including k-means, are underutilized, indicating that cluster analysis has not been extensively leveraged in this domain. Recent studies have emphasized energy-efficient and cluster-oriented optimization in cloud and edge computing. A threshold-based and cluster-centric scheduling strategies have been introduced to improve resource utilization and reliability^37–40^. This work proposes a hybrid Zk-means clustering approach that integrates Z-score standardization with centroid-based clustering to enhance robustness, interpretability, and considering energy efficiency in ramp event characterization. Unlike conventional frameworks where Z-score may be used solely as a preprocessing step for data normalization before k-means clustering in data analytics, while k-means clustering is used independently for pattern grouping, as summarized in Table 1, the proposed ZK-means framework redefines its role and embeds Z-score as a clustering-oriented analytical component that directly contributes to ramp severity discrimination and classification to influence distance measures and cluster formation, capturing both feature scaling and structural clustering advantages. This integrated design enables explicit ramp classification, improves physical interpretability, and unifies detection, clustering, and classification within a single low-complexity framework. Therefore, the novelty of the proposed method lies not in the individual techniques themselves, but in their hybridized analytical role and their systematic application in a domain-specific context like robust ramp event characterization, addressing a clear and previously unfilled research gap. The above analysis reveals a clear research gap in the literature. The proposed ZK-means framework directly addresses this gap. The key insights and research gaps are summarized belew:

1. Ramp event detection and characterization: Many methods focus on detection or forecasting of ramp events (e.g., wavelet, optimized swinging door algorithm (OpSDA) + deep learning).
  Research gap: There is limited research on robust categorization/clustering of identified ramp events into meaningful classes.
2. Threshold-based classification limitations: Thresholding techniques classify ramps, but often arbitrarily select thresholds, leading to sensitivity and heterogeneity.
  Research gap: A data-driven clustering framework that avoids arbitrary thresholds is needed.
3. Supervised vs. unsupervised methods: Supervised algorithms (e.g., gradient boosted trees) require labeled data, which is often scarce and subjective.
  Research gap: Unsupervised clustering approaches for ramp characterization are less explored, especially those that adapt to data distribution, feature scaling, and the presence of rare extreme ramp events.
4. General clustering usage: Conventional clustering (e.g., k-means, Ward) is applied mostly for wind speed patterns, not specifically for ramp event features (magnitude, duration, rate).
  Research gap: Need for specialized clustering techniques tailored to ramp attributes, beyond generic clustering usage.
5. Integration of preprocessing with clustering: While normalization and transformations (e.g., OpSDA, wavelets) enhance detection, integration with clustering methods is minimal in the literature.
  Research gap: Hybrid methods that integrate normalization effects as part of clustering logic (e.g., ZK- means) are largely unexplored.

**Table 1:** Comparative analysis of Z-score- and k-means-based approaches (2020–2025) and the proposed hybrid Zk-means framework.

Aspect	Existing literature (2020–2025)	Strengths (Pros)	Limitations/identified research gap	Proposed hybrid ZK-means framework
Role of Z-score	Z-score is mainly used for data normalization or statistical deviation analysis before learning or rule-based classification^22,41^	Simple, interpretable, and improves numerical stability	Z-score is not used as an explicit decision or clustering component	Z-score is elevated to an active analytical discriminator
k-means clustering usage	k-means applied for unsupervised grouping of renewable generation or electricity time-series patterns^42–44^	Computationally efficient; scalable	Clusters lack physical interpretation related to ramp severity	Cluster centroids explicitly mapped to distinct ramp behavior regimes
Z-score + k-means combination	Sequential preprocessing workflow: Z-score normalization followed by k-means clustering^41,44^	Improves clustering convergence and robustness	No co-decision or joint logic between Z-score and clustering	Introduces a true hybrid ZK-means architecture
Ramp event handling	Ramp detection via fixed thresholds, visualization, or segmentation, independent of clustering^31,45,46^	Effective basic detection	Limited adaptability; not cluster-aware	Ramp events are cluster-driven and adaptive
Ramp classification capability	Mainly binary or rule-based ramp severity classification^22,47^	Low computational burden	Lack of a structured multi-class taxonomy	Explicit multi-class ramp severity classification
Explainability	Implicit interpretability via statistical indicators or visualization^45,46^	Intuitive interpretation	Weak linkage between clusters and physical ramp behavior	Explainability via Z-score deviation logic and centroid semantics
Robustness to outliers	Outliers handled via filtering, smoothing, or segmentation^31,48^	Noise mitigation	Robustness handled externally to the core framework	Intrinsic robustness through hybrid statistical–cluster logic
Framework integration	Detection, clustering, and classification are treated as separate modules^45,47^	Modular and flexible	Lack of unified end-to-end methodology	Unified end-to-end ramp characterization framework

This paper introduces Zk-means to provide power system operators or planners with readily accessible information regarding anticipated ramp events. The main objectives are improving the accuracy, reliability, and interpretability of power ramp event characterization in the dominated renewable energy systems. The objectives can be summarized in the following points:

1. Addressing the limitations of existing ramp classification approaches (Z-score and k-means) by combining their strengths.
2. Using Z-score normalization to standardize the scale and decrease noise.
3. Improving ramp clustering efficiency using centroid-based partitioning in normalized space.
4. Providing interpretable mapping between statistical clusters (Z-space) and physical ramp levels (Δp), indicating low, moderate, high, and severe events.
5. Providing a flexible analytical framework that can be adapted to various datasets and renewable power scenarios.

The paper’s contribution can be summarized by the following points:

1. Introduces a new application for the k-means algorithm in clustering the power ramps. This approach is characterized by its speed, simplicity, generality, and ability to address the limitations of previous methods. It effectively avoids inconsistencies between various energy systems when defining the ramp event and introduces a cohesive framework for resolving this intricacy.
2. Introduces a novel application of the DBSCAN algorithm for analyzing and clustering ramp events in renewable generation. To the best of our knowledge, this is the first study to employ DBSCAN for ramp event characterization. This addition provides new insights into density-based clustering performance under variable data conditions and highlights both the strengths and limitations of DBSCAN when compared with the proposed ZK-means method.
3. Introduces the ZK-means algorithm, an innovative hybrid method for ramp categorization. This method integrates k-means clustering with Z-scores, which are utilized as part of clustering logic to optimize clustering efficacy, enhance convergence stability, improve interpretability, and guarantee consistent classification of ramp events across multi-year datasets.
4. Presents a decade-long actual case study illustrating that the ZK-means method exhibits enhanced stability and reduced within-cluster variance relative to traditional k-means, Z-score, and DBSCAN algorithms, thereby affirming its efficacy for extensive, high-dimensional renewable energy datasets.
5. Provides pragmatic insights for grid operators and system planners by matching clustering results with operational ramp management strategies, thus enhancing the comprehensibility of ramp dynamics for decision-making in renewable-dominant power systems.
6. The techniques applied unveiled the almost consistent relative frequency of each ramp category.
7. To accurately represent the power ramps in the system, the number of clusters can be increased when there are high standard deviation values. This is because the magnitudes of the power ramps cover a wider range.
8. The classification technique’s suitability for various time intervals.

## Methodologies under consideration

**Fig. 1:**
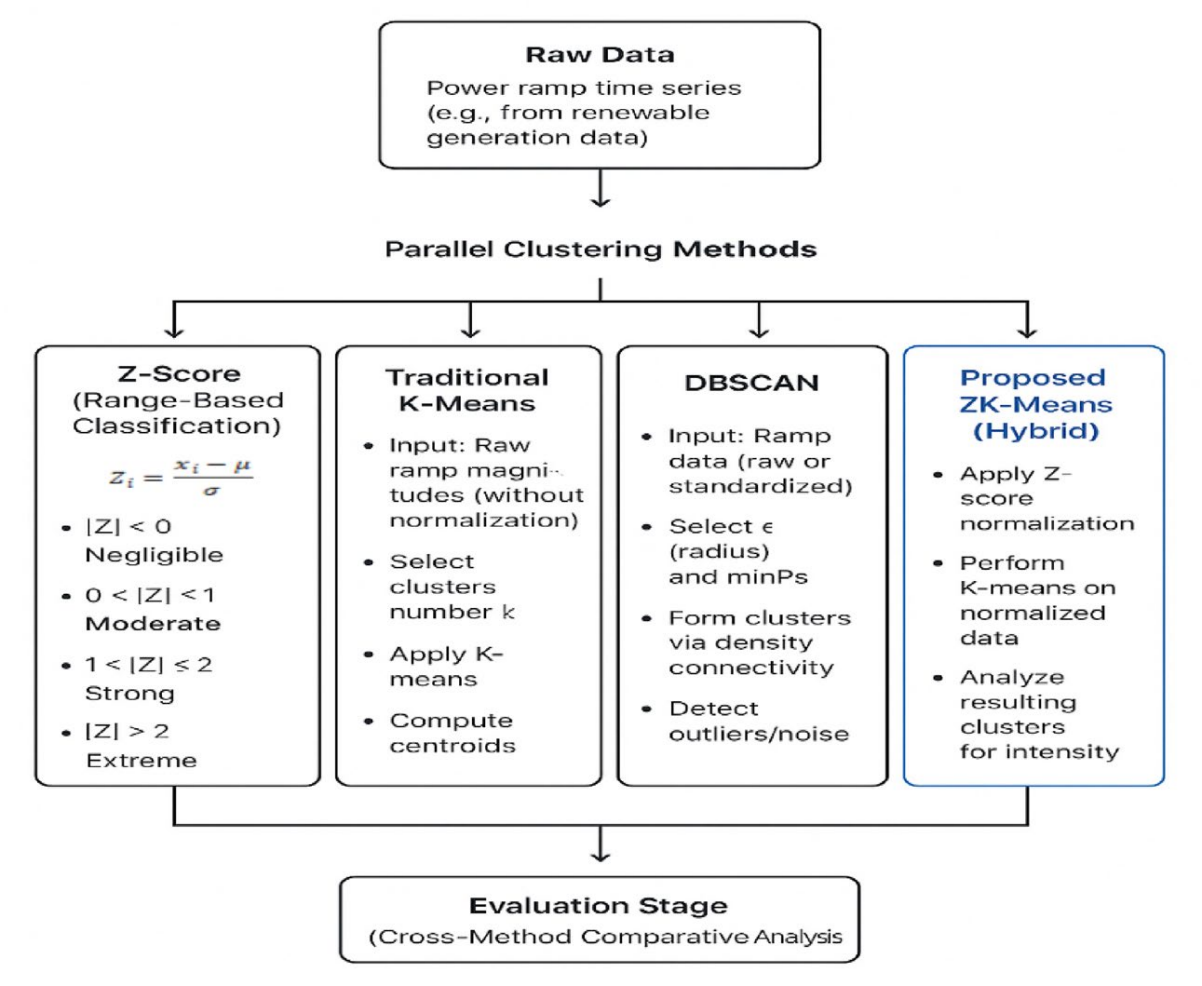
Comparative workflow of the proposed ZK-means method versus Z-score, traditional k-means, and DBSCAN clustering approaches.

Clustering techniques are used to group similar data points into clusters. The goal is to maximize similarity within each cluster while minimizing similarity between different clusters. The proposed method, Zk-means, combines two clustering methods. The first is the Z-score, which was recently published in ^22^ and uses Z-scores for ramp event classification, and the second method is the k-means algorithm. Although k-means clustering was widely applied in various fields, including wind power, it was not used for ramp event classification. There have been two phases of comparison between the proposed ZK-means approach and current clustering algorithms. At first, the suggested hybrid approach was compared against the methods that make it up to see how much value they provided by being combined. Then, it was compared to an alternative clustering method, density-based spatial clustering (DBSCAN), to ensure its robustness and general applicability. A unified workflow has been developed, as illustrated in Fig. 1. The framework begins with the preprocessing of raw power data (data cleaning, missing value handling, ramp detection) and proceeds through four parallel analytical branches representing the Z-score classification, traditional k-means, DBSCAN, and the proposed ZK-means approach, where each method operates independently on the same input dataset and produces clusters based on its underlying principle. The evaluation stage then consolidates the results through comparative analysis, focusing on clustering principle, parameter sensitivity, outlier handling, interpretability, scalability, and suitability for long-term trend analysis.

Let *X* = {*x*_1_,*x*_2_,…,*x*_*n*_} represent the dataset of power ramps**,** where each data point *x*_*i*_ = *Δp*_*t*_ denotes the change in power output between consecutive time intervals, which is calculated by Eq. (1). The power ramps are first separated into upward and downward types. The objective is to cluster the ramp magnitudes into meaningful clusters that reflect their severity. In the next subsection, the three methods will be illustrated in detail.

1 $$\Delta p_{t} = P\left( {t + \Delta t} \right){-}P\left( t \right)$$

### Z-score classification method (range-based)

This methodology is interpreted as a two-step process:

1. Z-score standardization: The power ramps are transformed into Z-scores to have a mean of zero and a standard deviation of one, effectively normalizing feature scales.
2. Range-Based grouping: After standardization, data points are directly assigned to predefined *k* clusters based on pre-determined ranges of their Z-score values. This method utilizes scaled values to directly delineate cluster boundaries without iterative optimization. The two steps are illustrated below in detail:

In VRG, such as wind generation, due to continuous fluctuations, the average power ramp is often near zero ^49,50^. So, historical power ramps are first separated into upward and downward types. For each type, the average value and standard deviation are calculated. Each power ramp is then assigned a Z-score, which indicates how many standard deviations its magnitude is above or below the average. This transforms the data into standard deviation units, where the average has a score of zero and one standard deviation has a score of one. Equation (2) illustrates the concept of Z-scores by showing how specific data points relate to the mean and standard deviation.

2 $${Z}_{i}=\frac{{x}_{i}-\mu }{\sigma }$$

Here $x$ is the data point representing the power ramp ($\Delta p$), $\mu$ is the mean of the data representing the average power ramp value (${\Delta p}_{avg}$), and $\sigma$ is the standard deviation of the ramp data, which can be reformulated as shown in Eq. (3).

3 $${Z}_{i}=\frac{{\Delta p}_{i}-{\Delta p}_{avg}}{\sigma }$$

Finally, these scores are used to classify power ramps into k clusters based on pre-determined Z-score ranges, as can be represented by Eq. (4).

4 $${C}_{j}=\left\{{\Delta p}_{i}\left|{\alpha}_{j}\le \left|{Z}_{i}\right|<{\beta}_{j}\right.\right\}$$

where ${\alpha}_{j}$ and ${\beta}_{j}$ denote the lower and upper Z-score thresholds defining the *j*th ramp category. In the initial application of this methodology^22^, four levels were suggested: low, moderate, high, and severe, as summarized in Table 2.

**Table 2:** Four-category classification of power ramps based on Z-scores.

Ramp category	Constraint	Description
Low	$\left|{Z}_{i}\right|<0$	Negligible or no ramp
Medium	$0<\left|{Z}_{i}\right|\le 1$	Moderate ramp variation
High	$1<\left|{Z}_{i}\right|\le 2$	Noticeable or strong ramp fluctuation
Severe	$\left|{Z}_{i}\right|>2$	Extreme ramp event

#### Applications of Z-score clustering

The Z-score clustering technique has proven to be highly effective for categorizing ramp events in both wind and photovoltaic data. It enables the systematic categorization of these variations into specific levels, generally defined as low, moderate, high, and severe ^22,51^. This thorough classification is crucial for grid operators, enabling them to enhance their understanding, predict, and manage the effects of rapid fluctuations in VRG. The Z-score method standardizes data points based on their deviation from the mean, providing a normalized metric that considers the intrinsic variability of diverse datasets. This facilitates a uniform and comparable evaluation of ramp severity, regardless of the particular climatic attributes or temporal resolution of the gathered data. The capability to distinguish between these severity levels is invaluable for:

- Grid stability: Identifying high and severe power ramps helps in anticipating periods of significant power imbalance, allowing for proactive measures to maintain grid stability.
- Energy storage optimization: The understanding of ramp magnitudes and frequencies supports the optimal sizing and dispatch of energy storage systems that are essential for smoothing out renewable power variations.
- Forecasting improvement, as the detailed ramp classification can refine forecasting models, leading to more accurate predictions of power output variability and enhancing day-ahead and intra-hour operational planning.
- Market operations: A better characterization of ramps supports more efficient energy market operations by reducing uncertainty associated with the integration of VRG.

The Z-score classification technique, originally evaluated for wind power data and later generalized to solar power data, underscores its potential as a broadly applicable technique in the studies of renewable energy and other fields requiring robust anomaly detection and data categorization.

### K-means clustering (traditional)

**Fig. 2:**
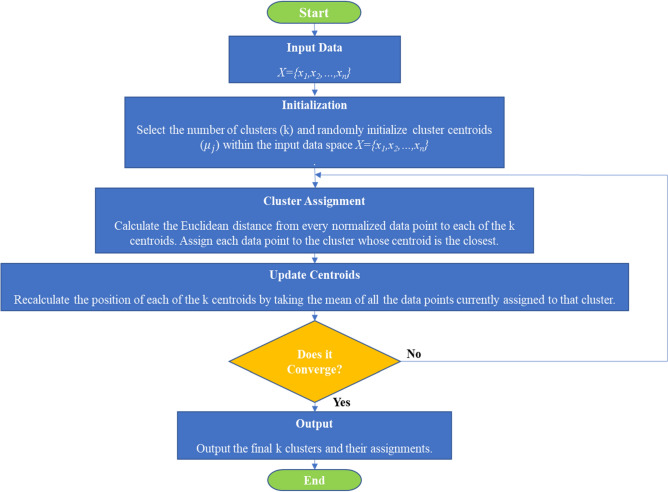
The flowchart of k-means.

Traditional means the direct application of the k-means algorithm to the raw data, unscaled wind power data. The k-means technique seeks to partition *n* observations into *k* clusters, assigning each observation to the cluster with the nearest centroid. The k-means algorithm is a widely used unsupervised machine learning method characterized by its simplicity and ease of implementation, making it one of the fastest clustering techniques. The k-means algorithm is versatile in its capability to handle different types of features, such as numeric and binary. It is capable of efficiently handling big data sets by grouping data points into distinct clusters based on their similarities, with each cluster being represented by its centroid, which is the average of all data points assigned to that cluster. It requires a predetermined cluster number. The input data is the row data and the number of clusters. The objective of k-means is to minimize the variance within each cluster by minimizing the sum of squared distances between each data point and its assigned centroid within each cluster by iteratively moving the centroids to places closer to them than to any other centroid. k-means clustering harnesses the capabilities of machine learning and data analytics. Using the Euclidean distance, the algorithm assigns each data point to its closest center, which is calculated by Eq. (5).

5 $${\mu}_{j}=\frac{1}{{N}_{j}}\sum_{i=1}^{{N}_{j}}{x}_{i}^{j}$$

where ${\mu}_{j}$ denotes the mean value of the data points assigned to cluster *j*, ${x}_{i}^{j}$ is the *i*-th data in the cluster $j$, and *N*_*j*_ is the number of data points in the cluster $j$. The main idea is to obtain optimal centroids for each of the assumed k clusters; this is done by assuming initial centroid locations and then iterating to identify the optimal locations. The iteration process is terminated once the objective function ($J$) achieves its minimum to an agreed resolution. $J$ is defined by Eq. (6):

6 $$J= \sum_{j=1}^{k}\sum_{i=1}^{{N}_{j}}{\Vert {x}_{i}^{(j)}- {\mu}_{j}\Vert }^{2}$$

where $J$ denotes the within-cluster sum of squares (WCSS), which quantifies the total squared distance between all points and their respective cluster centroids, and ${\Vert {x}_{i}^{(j)}- {\mu}_{j}\Vert }^{2}$ is the squared Euclidean distance between data points ${x}_{i}^{(j)}$ and its cluster center ${\mu}_{j}$. The ramp data is partitioned into $k$ clusters, where the data points within each cluster exhibit a high degree of similarity. The quality of the data significantly influences the performance of k-means clustering, as this approach is based on Euclidean distance; it is susceptible to being influenced by outliers. A small number of outliers might distort the centroids, leading to possibly inaccurate cluster formations. Moreover, the main obstacle associated with the k-means algorithm is the need to pre-determine the optimal number of clusters (k), as it is not automatically determined ^52^. The k-means method may experience suboptimal convergence and inferior clustering outcomes because of its random startup and sensitivity to beginning conditions (the first selection of centroids). Furthermore, it has a tendency to converge towards local minima rather than discovering the global optimum ^20,53,54^. The flowchart of k-means is presented in Fig. 2. The clustering algorithm possesses the primary sequential stages as follows:

1. The k objects are selected as the initial clustering centers from the dataset that contains N objects.
2. The nearest cluster is determined by computing the distance value between objects and the central points of clusters.
3. The cluster’s mean value is computed in a continuous manner, and the centroid of the cluster is updated during the iteration.
4. Steps 2 and 3 are iteratively performed until the center of each cluster is no longer updated.

The result is highly sensitive to the choice of the number of clusters *k*, and it is often not evident how many clusters to choose. This becomes particularly difficult when managing complex multi-dimensional power system data. Diverse methodologies can be employed to ascertain the optimal number of clusters, including the “elbow” method and the silhouette score ^55,56^. This underscores the importance of integrating reliable statistical methods to achieve optimal results.

#### Determining the optimal number of clusters in k-means clustering

Picking the appropriate number of clusters (*k*) is a critical step in k-means clustering. Two widely used methods for this purpose are the elbow method and the silhouette score ^57^.

1. Elbow method: It examines the trend of WCSS as the number of clusters increases. WCSS measures the sum of squared distances between each point and the centroid of its assigned cluster. As *k* increases, WCSS decreases because data points are nearer to their corresponding cluster centroids. When plotting WCSS against *k*, the optimal value of* k* is often identified at a kink or elbow in the graph. This point indicates where the rate of decrease in WCSS significantly slows down, implying that incorporating additional clusters beyond this point provides diminishing returns in terms of reducing within-cluster variance.
2. Silhouette score: It quantifies how similar an object is to its cluster compared to other clusters. This dimensionless score ranges from − 1 to 1. It is calculated for each data point in each cluster based on two values (a, b). In turn, the Silhouette score for a single data point is estimated as in Eq. (7):
  7 $$Silhouette Score=\frac{b-a}{max(a,b)}$$

where $a$ is the average distance between a single data point and all other data points in the same cluster, $b$ is the average distance between that data point and the next closest cluster’s other data points, and $max(a,b)$ refers to selecting the maximum value between *a* and *b*. The high value of Silhouette score (close to 1) indicates that the sample is well-matched to its cluster and poorly matched to neighboring clusters, suggesting a distinct cluster, and the value around zero suggests that the sample is on or very close to the decision boundary between two clusters, while the negative value (close to -1) indicates that the sample might be assigned to the wrong cluster. The overall silhouette score for a clustering model is its average value for all the data points. This offers a unique metric for assessing the overall quality of the clustering solution. A high overall score indicates that the clusters are distinctly separated. An inadequate overall score may indicate overlapping clusters or a suboptimal selection of the number of clusters. An evaluation of the silhouette score across varying *k* values typically reveals that a declining trend with increasing *k* suggests a preference for a smaller number of clusters to preserve robust cluster cohesiveness. This method evaluates both the density of clusters and their inter-cluster separation.

#### Applications of k-means clustering

**Fig. 3:**
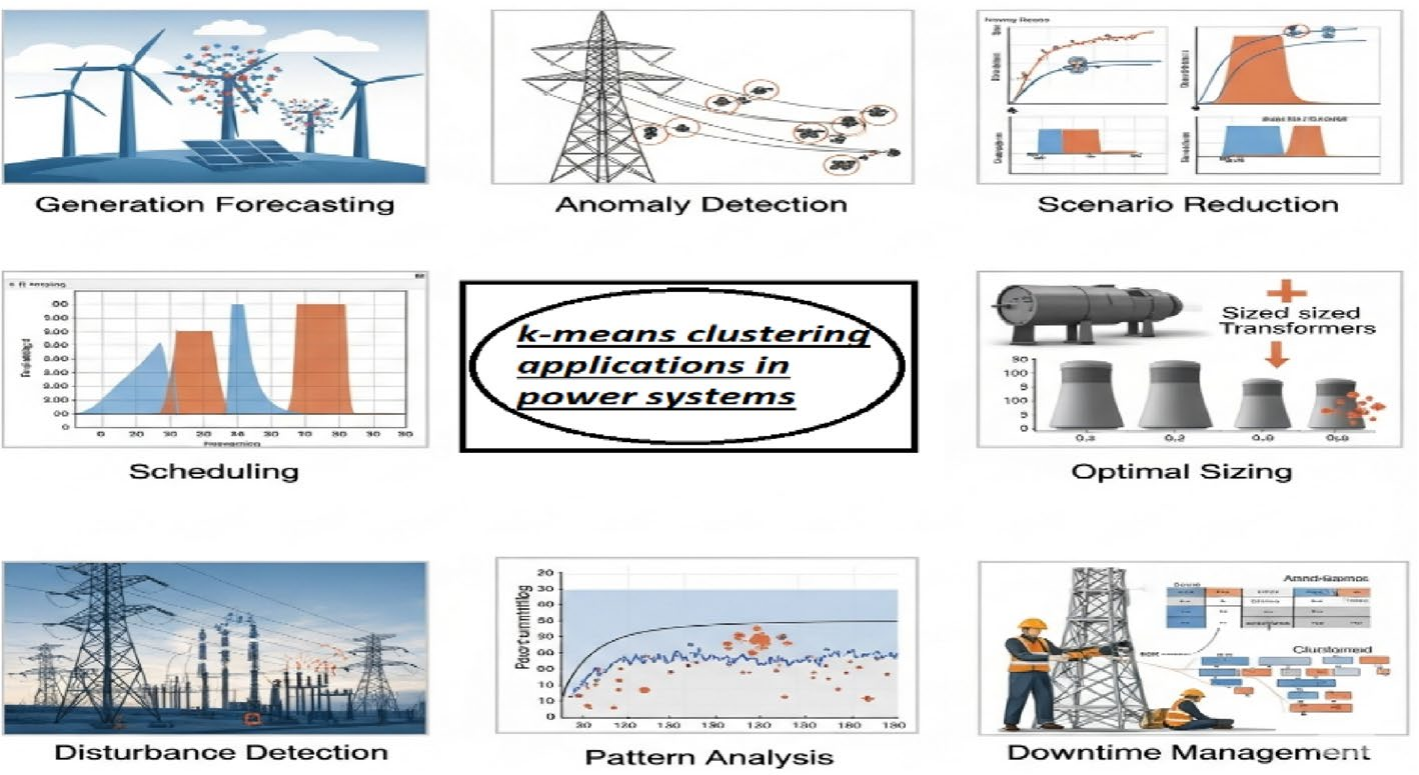
Diverse applications of k-means clustering in power systems.

The k-means heuristic is widely used across the sciences, with applications spanning genetics, image segmentation, customer segmentation ^58^, grouping search results and news aggregation, crime-hot-spot detection, crime pattern analysis, and profiling road accident hot spots. k-means clustering has been used in modern power systems to decrease the overall number of inspection teams assigned to the maintenance of transmission lines and to generate inspection pathways that are nearly ideal and distribute labour hours evenly among each team ^59^. It has been employed to ascertain the best location for the data aggregation point in order to gather data from smart meters ^60^. The incorporation of plug-in hybrid electric vehicles might provide difficulties for the electric grid as a result of the uncertain nature of their connections. By employing k-means clustering, it is possible to categorize them into separate fleets, thus reducing this problem ^61,62^, optimizing the anticipated incremental revenue of the distribution system operator ^63^, and optimizing the allocation of charging stations ^64,65^. The k-means algorithm has been used to detect near misses inside secure situations as it enables the timely recovery of failure combinations, hence preventing accidents, minimizing system downtime, and decreasing associated risks ^66^. A proposal for the optimal size of storage systems was introduced in ^67^. After clustering the load profile using k-means, a bi-level optimization is conducted that takes into account both minimizing costs and reducing power deviation. The k-means algorithm is employed in renewable generation with distributed renewable energy sources to address generation balance and scenario selection ^68,69^. This involves preserving simultaneous and chronological combinations of various loads and distributed energy supplies. k-means clustering is commonly employed to reduce scenarios, simplifying and directing the study towards crucial patterns and trends present in the data ^70,71^. It has also been employed in resource assessment ^57,72^, site and size selection ^72–75^, real power loss reduction ^76^, demand forecasting ^77^, load profiling ^58,78^, renewable power prediction ^79–81^, power quality analysis ^82^, fault detection and diagnosis ^83–87^, system security assessment and segmentation of the energy market by clustering renewable energy producers based on their generation characteristics and geographic locations. It helps identify patterns in renewable resources and geographical features, improving resource allocation and prioritizing sites ^72^. It also aids in customizing renewable generation strategies based on consumer usage patterns. It can aid in identifying spatial and temporal correlations in renewable generation patterns ^68^, allowing for the optimization of grid operation strategies. By clustering regions with similar generation profiles, system operators can implement tailored control strategies, such as demand response and energy storage management, to mitigate the impacts of variability in renewable generation, which helps in aligning supply and demand, ensuring grid stability, and promoting the integration of VRG into the grid. In ^88,89^, the k-means clustering algorithm was utilized to enhance the accuracy of wind power forecasting. It has been demonstrated that the k-means clustering algorithms outperform other cluster-based methods currently in use. Further research is needed for k-means clustering to maximize its benefits for power system optimization and renewable energy integration. In ^90^, the wind speed prediction was done using two distinct techniques: k-means clustering and Mycielski-3. The k-means clusters produced a better prediction result than Mycielski-3. In ^91^, the k-means clustering algorithm was utilized to predict the PV power output and gave high prediction accuracy. Figure 3 illustrates the various applications of k-means clustering in power systems, particularly in renewable generation, which provides several benefits, including:

- Enhanced resource utilization and optimized energy production efficiency.
- Clustering facilitates a more effective understanding and management of distributed energy resources, resulting in enhanced grid stability and flexibility in the face of output power variations from VRG, improving grid integration and advancing the transition towards a sustainable and low-carbon future.
- Clustering also reduces operational costs and enhances the economic viability of renewable energy projects.

### The proposed Zk-means clustering approach

The Zk-means approach is introduced, integrating the statistical robustness of z-score normalization with the clustering efficiency of iterative centroid optimization (k-means) in a unified framework with global Z-score application and yearly independent clustering, utilizing a more creative starting technique, as each ramp is first standardized as discussed earlier to eliminate scale dependency and suppress the influence of outliers. Then, k-means clustering is performed to minimize the WCSS:

8 $${J}_{ZK}= \sum_{j=1}^{k}\sum_{i=1}^{{N}_{j}}{\Vert {z}_{i}^{\left(j\right)}- {\mu}_{j}\Vert }^{2}$$

Here, ${\Vert {z}_{i}^{(j)}- {\mu}_{j}\Vert }^{2}$ denotes the squared Euclidean distance between the normalized ramp and its assigned centroid. Hence, this methodology entails two steps:

1. Z-score standardization: This transforms the data into standard deviation units.
2. k-means application: The k-means algorithm is subsequently applied to the transformed data to perform the final clustering.

**Fig. 4:**
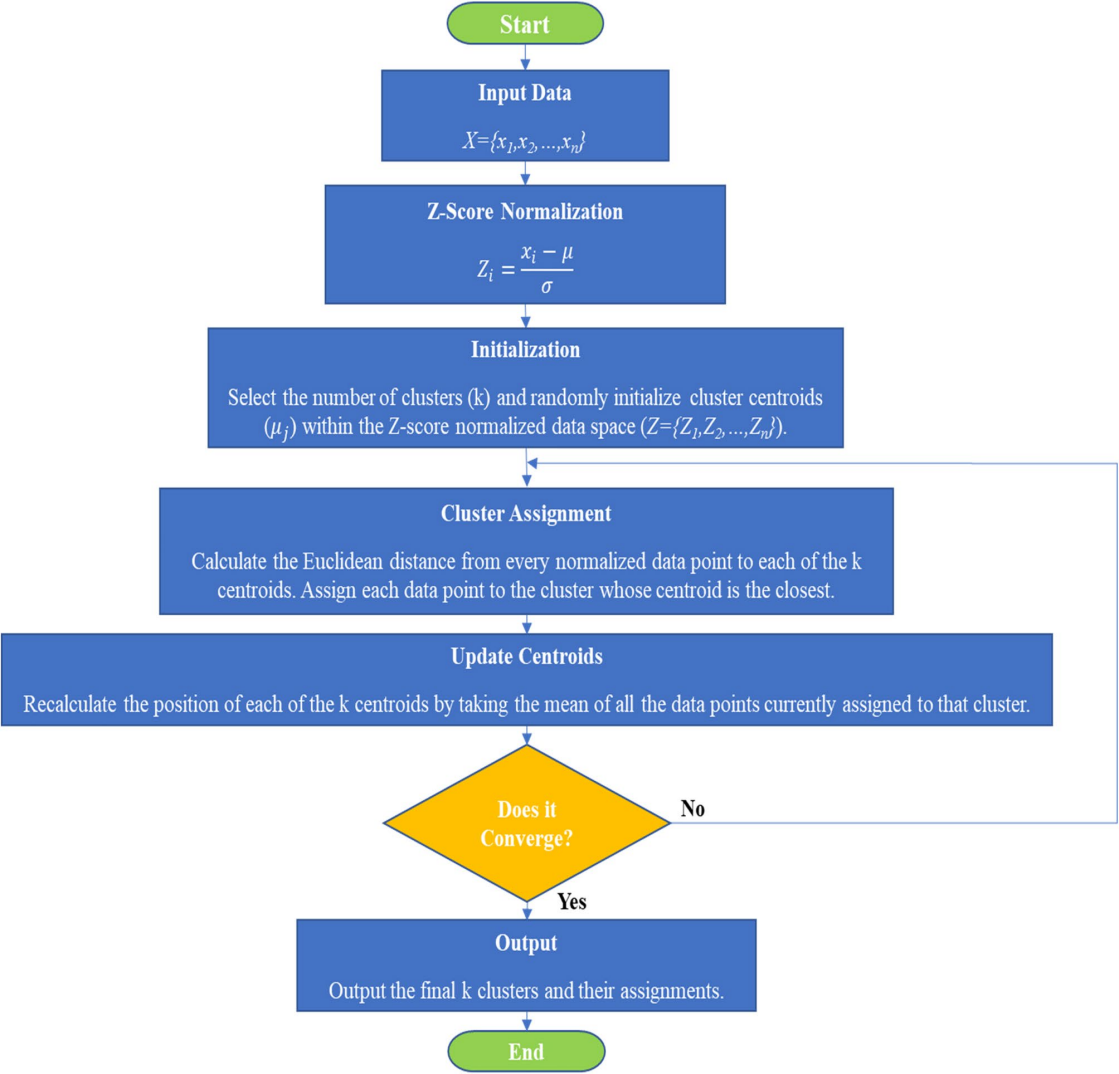
Zk-means flowchart.

**Fig. 5:**
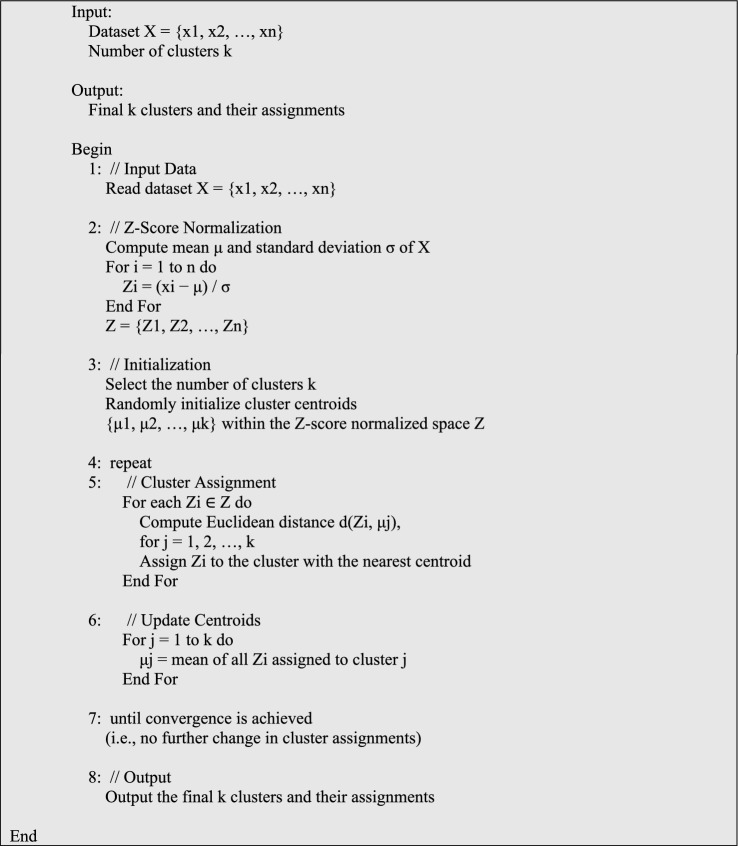
The pseudocode of ZK-means approach.

**Fig. 6:**
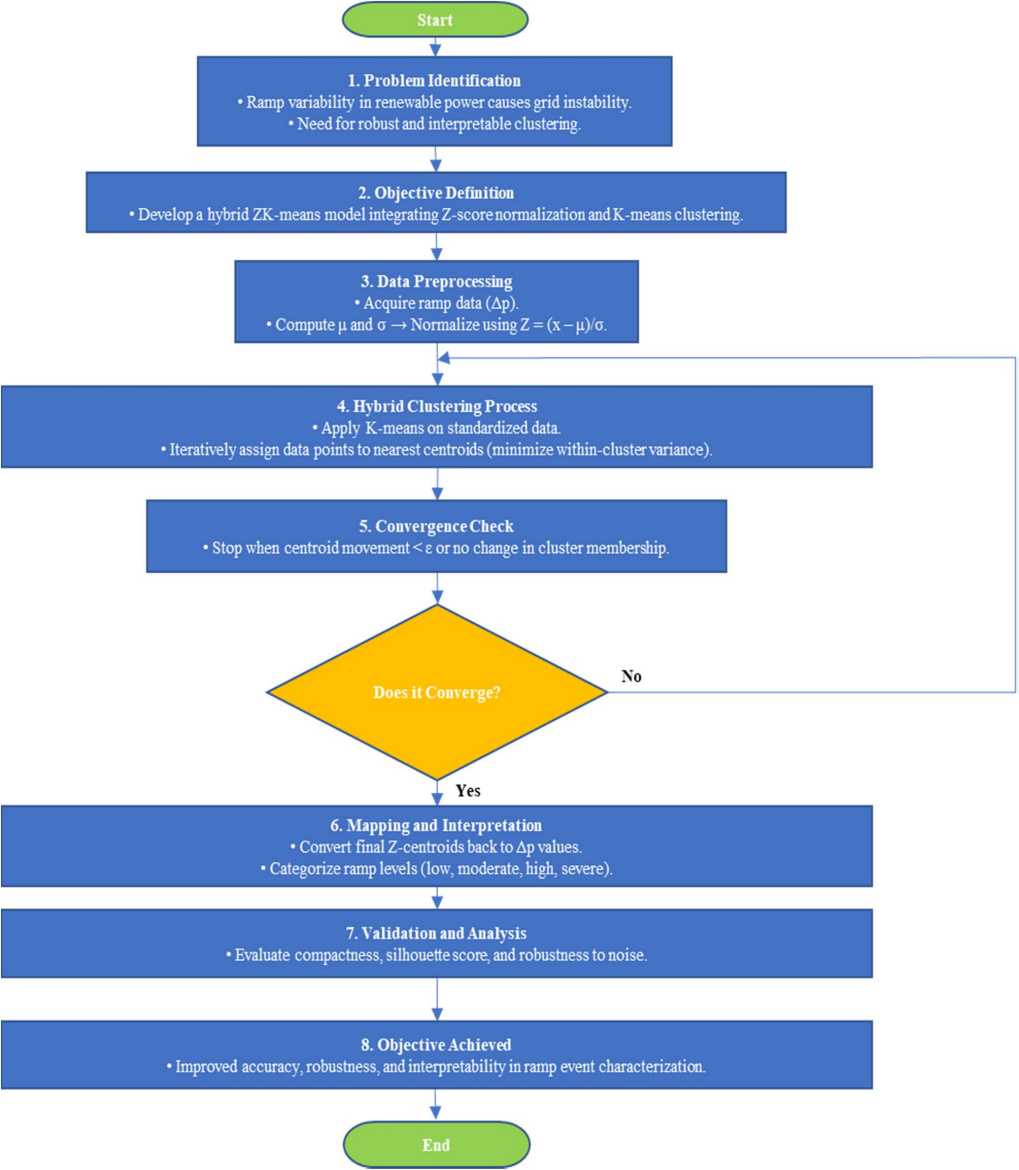
Flowchart of objective–methodology relationship.

The flowchart of Zk-means is presented in Fig. 4, and the pseudocode corresponding to the ZK-means flowchart is illustrated in Fig. 5. This hybrid formulation ensures a consistent and balanced treatment of power ramp magnitudes by combining the statistical normalization capability of the Z-score transformation with the clustering efficiency of the k-means algorithm. As a result, the ZK-means method provides enhanced robustness to noise, since clustering is performed in the standardized Z-space, improved cluster compactness as Z-score normalization minimizes the distortion caused by large ramp magnitudes, and greater interpretability in power system ramp analysis, because cluster centroids in Z-space can be mapped back to actual power ramp levels (Δ*p*) corresponding to low, moderate, or severe events. Moreover, the method exhibits higher flexibility, enabling adaptive determination of cluster boundaries based on both statistical deviations and spatial proximity among ramp events. To ensure the methodology directly satisfies these objectives, the proposed ZK-means process is designed as a goal-oriented sequence, as outlined in Table 3 and shown schematically in Fig. 6.

**Table 3:** Methodology–objective alignment.

Objective	Methodological action	Outcome/constraint satisfied
1. Combine statistical and clustering advantages	Apply Z-score normalization followed by k-means clustering	Integrates statistical robustness with efficient partitioning
2. Ensure consistent scaling and outlier resistance	Use standardized Z-space to eliminate scale dependency	Reduces distortion from large ramps and suppresses noise influence
3. Achieve compact, meaningful ramp clusters	Apply k-means clustering on Z-score normalized data to minimize within-cluster variance	Z-score ensures uniform scaling; k-means produces compact, well-separated ramp groups
4. Maintain interpretability of ramp categories	Map Z-space centroids back to physical Δp values	Enables direct and intuitive understanding of ramp severity levels
5. Adapt to diverse ramp profiles	Determine optimal k dynamically based on Z-score distribution and clustering validity indices	Ensures flexibility and adaptability across different datasets without relying on fixed thresholds

### Code availability

The methodology proposed in this paper has been fully described through the mathematical equations, algorithmic steps, and workflow presented in the manuscript. The clustering analysis was implemented using XLSTAT Premium Software; see further details in “XLSTAT premium software”. No proprietary custom code was developed beyond the analytical framework described in the article. Therefore, no separate code repository is required. Readers can reproduce the method using the equations, algorithmic procedure, and software settings reported in the manuscript. Additional implementation details can be obtained from the author upon reasonable request.

## Case study (Belgium’s output of wind power)

**Fig. 7:**
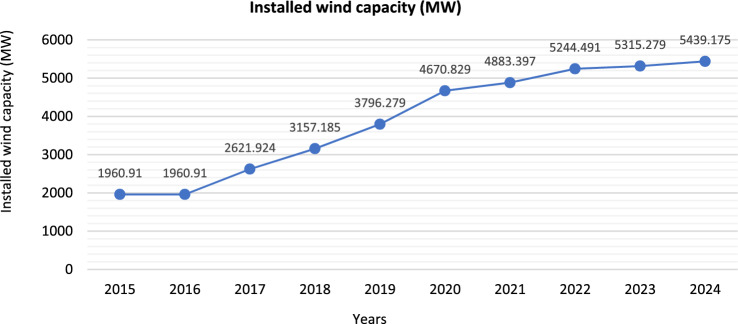
Yearly installation of Belgium’s wind capacity in MW.

The installation of wind capacity in Belgium is growing continuously, as shown in Fig. 7. At the end of 2015, the maximum installed wind capacity was 1960.91 MW. Whereas in 2024, the maximum installed wind capacity was 5439.175 MW. The output power from aggregated wind farms (onshore and offshore) is recorded every 15 min. During the specified time interval (Δt = 15 min), the fluctuations in wind power from Belgium’s aggregated wind farms over 10 years from 2015 to 2024 will be determined, and the power ramps will be classified for patterns in wind power variation. All computations were carried out on a PC using XLSTAT Premium software, equipped with an Intel® Core™ i7-7700 CPU @ 3.60 GHz and 16 GB RAM. This is done to better understand, predict, and manage the impact of wind power’s natural variability on the stability of the electrical grid, evaluating ZK-means against its constituent methods to assess the benefits of their combination. Then, it is compared to the DBSCAN algorithm to verify its robustness and general applicability. The initial dataset may include missing values or values that are not logically possible, such as wind power values that are below zero. This data must be deleted. Subsequently, it is imperative to address the void left by the eradicated and absent data; thus, the Nearest Neighbor approach is utilized. After that, the initial dataset is divided into upward and downward ramps, and then for each subset, the classification techniques are applied. The summary statistics for upward and downward ramps in ten years, including for each year, the number of ramps (Observations), the minimum and maximum magnitude, and the standard deviation, are presented in Table 4.

**Table 4:** Summary statistics for upward and downward ramps.

Year	Upward ramps					Downward ramps				
	Observations	Minimum (MW)	Maximum (MW)	Mean (MW)	$\sigma$ (MW)	Observations	Minimum (MW)	Maximum (MW)	Mean (MW)	$\sigma$ (MW)
2015	17,280	0.010	361.080	20.598	23.321	17,744	0.010	300.590	20.038	22.457
2016	17,451	0.010	266.310	19.600	22.243	17,672	0.010	319.790	19.391	22.625
2017	17,274	0.010	444.040	24.032	28.278	17,741	0.010	654.840	23.317	28.059
2018	17,341	0.010	736.930	27.887	32.741	17,694	0.010	455.860	27.383	31.440
2019	17,387	0.010	650.960	37.388	44.918	17,646	0.010	692.090	36.839	43.698
2020	17,436	0.010	615.170	41.407	50.781	17,684	0.020	737.580	40.858	49.960
2021	17,317	0.010	879.860	47.059	60.209	17,715	0.010	1038.800	45.883	58.534
2022	17,426	0.010	1271.64	50.208	66.551	17,607	0.010	1175.110	49.574	66.547
2023	17,247	0.010	2521.397	55.554	75.015	17,788	0.010	2530.497	53.871	72.27
2024	17,243	0.010	2250.352	51.412	69.228	17,886	0.010	2232.632	49.582	66.594

### XLSTAT premium software

To ensure reliable, reproducible, and efficient execution of the proposed methodology, all clustering analyses were performed using XLSTAT Premium (Network version 2024.1.1), a comprehensive statistical analysis add-in for Microsoft Excel that provides a wide range of data preprocessing, statistical modeling, and machine learning tools through a user-friendly graphical interface. Its seamless integration with Excel enables efficient data handling, reproducibility, and direct export of analytical results for further processing and visualization. The dataset was first imported into XLSTAT and standardized via Z-score normalization, followed by applying the k-means clustering tool within XLSTAT to the normalized dataset, where the number of clusters *k* was predefined. Euclidean distance was selected as the similarity measure, and centroid initialization was performed randomly within the normalized feature space. The algorithm iterated until cluster assignments converged, and the resulting clusters and centroids were used for result visualization and performance analysis.

## Results and discussion

### Z-score classification results

**Fig. 8:**
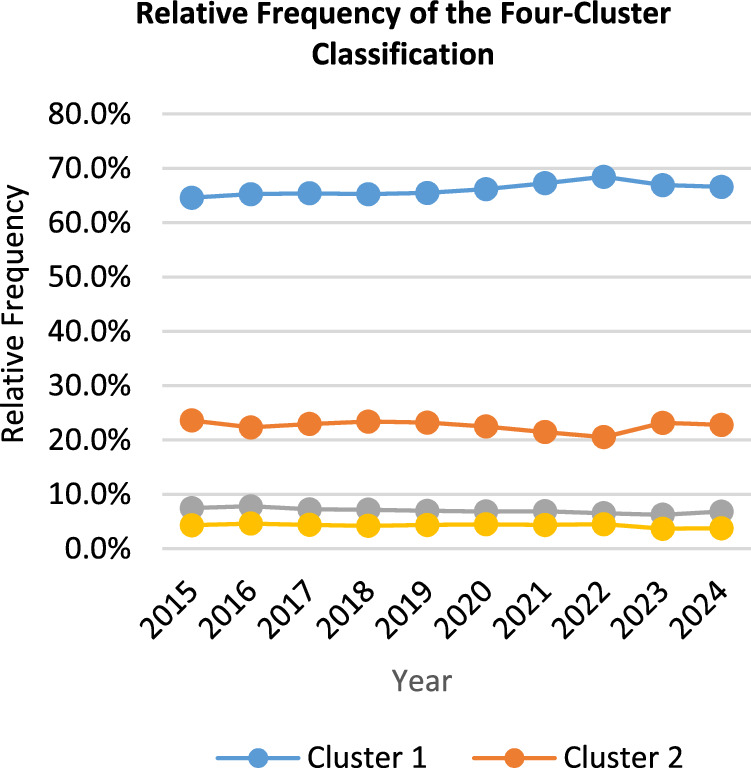
Four-category classification for upward ramps based on Z-scores.

**Fig. 9:**
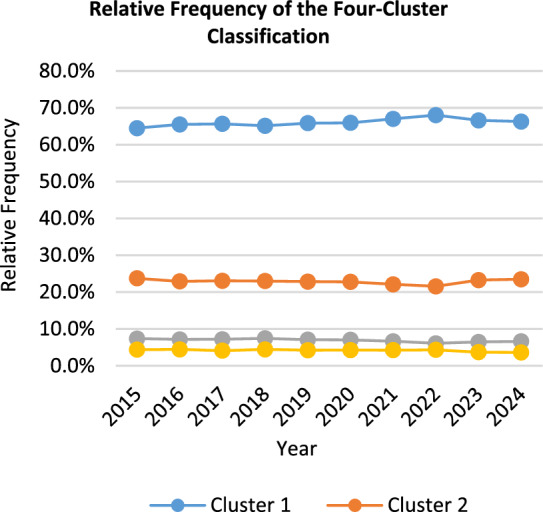
Four-category classification for downward ramps based on Z-scores.

The classification results illustrated the yearly features associated with wind power ramps. Figures 8 and 9 show that the 10 years have nearly a fixed percentage for each ramp category, and the relative frequency of both types of ramps is the same. For upward power ramps, the relative frequency of low power ramps (cluster 1) ranges from 64.6% to 68.5%, medium power ramps (cluster 2) ranges from 20.5% to 23.6%, high power ramps (cluster 3) ranges from 6.2% to 7.8%, and severe power ramps (cluster 4) ranges from 3.7% to 4.6% of the total power ramps. For downward power ramps, the relative frequency of low power ramps ranges from 64.5% to 68%, medium power ramps ranges from 21.6% to 23.7%, high power ramps ranges from 6.1% to 7.4%, and severe power ramps ranges from 3.6% to 4.4% of the total power ramps. These findings align with previous research using ramp characteristic indicators (RCI) ^92,93^, which also demonstrated that the class boundaries, determined by the average and standard deviation of power ramps, maintain a consistent percentage of the average wind installation, yielding a stable relative frequency for each category.

### k-means classification results

The classification results illustrated the yearly features associated with wind power ramps. In the next subsections, the classification results for the four cluster classifications and the optimal cluster number based on the Silhouette score evaluation for both ramp types are presented.

#### Four cluster classification for upward ramps by k-means

**Fig. 10:**
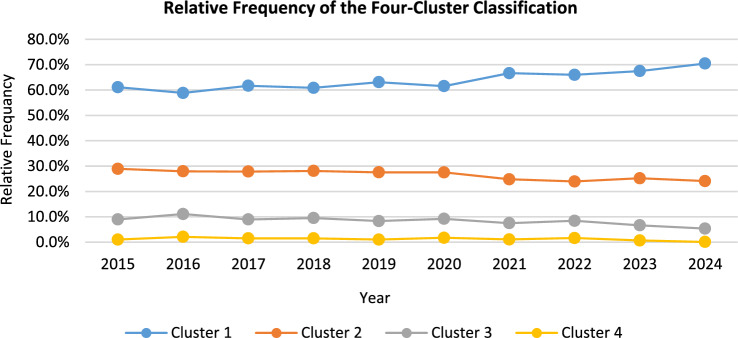
Four cluster classification for upward ramps by k-means.

Table 5 presents the classification results and summarizes the characteristics of each cluster over the ten years. Figure 10 shows that the 10 years have nearly a fixed percentage for each ramp category, as the relative frequency of low power ramps (cluster 1) ranges from 58.8% to 70.5%, medium power ramps (cluster 2) ranges from 23.9% to 28.9%, high power ramps (cluster 3) ranges from 5.4% to 11.1%, and severe power ramps (cluster 4) ranges from 0.1% to 2.1% of the total power ramps.

**Table 5:** Four cluster classification for upward ramps by k-means.

Cluster data		Year									
		2015	2016	2017	2018	2019	2020	2021	2022	2023	2024
Cluster 1	Centroid (MW)	7.605	6.537	8.551	9.968	13.696	14.022	17.298	17.439	21.473	20.956
	N of ramps	10,559	10,268	10,659	10,556	10,973	10,733	11,541	11,503	11,641	12,149
	Within-cluster variance	28.365	21.04	36.257	48.070	97.133	105.75	169.456	172.88	257.663	267.273
	Silhouette scores	0.690	0.696	0.701	0.694	0.698	0.697	0.711	0.709	0.706	0.721
Cluster 2	Centroid (MW)	29.753	25.96	34.445	39.131	55.621	57.774	74.952	75.391	91.542	95.614
	N of ramps	4999	4878	4810	4874	4788	4796	4294	4173	4346	4154
	Within-cluster variance	62.557	46.80	87.03	106.34	232.64	247.28	454.64	454.66	662.3	801.68
	Silhouette scores	0.510	0.502	0.502	0.508	0.509	0.513	0.498	0.501	0.514	0.503
Cluster 3	Centroid (MW)	65.387	55.062	75.533	84.368	125.714	130.255	171.731	172.85	215.278	234.6
	N of ramps	1547	1937	1547	1650	1448	1604	1297	1468	1144	924
	Within-cluster variance	212.2	129.9	278.01	334.25	800.41	833.17	1526.3	1464.74	2847.62	5187.92
	Silhouette scores	0.493	0.496	0.483	0.497	0.502	0.494	0.493	0.499	0.488	0.453
Cluster 4	Centroid (MW)	147.14	113.1	160.69	185.58	288.91	282.08	382.22	375.82	525.13	1122.79
	N of ramps	175	368	258	261	178	303	185	282	116	16
	Within-cluster variance	2011.75	909.16	2257.63	4813.06	8090.79	5815.99	12,951.75	14,819.46	95,441.85	167,318.5
	Silhouette scores	0.434	0.468	0.449	0.394	0.429	0.450	0.420	0.449	0.327	0.435

**Fig. 11:**
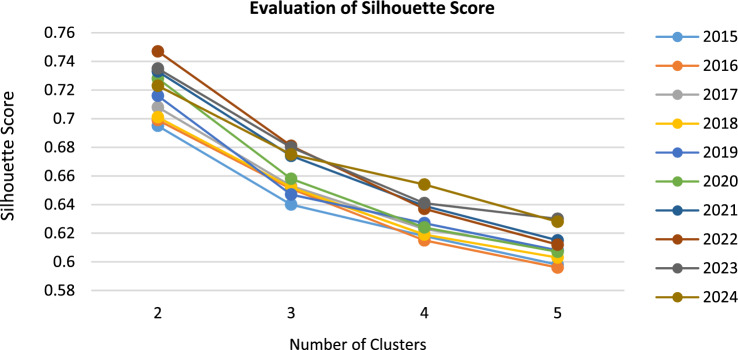
Optimal number of clusters for upward ramps over ten years based on Silhouette score evaluation.

**Fig. 12:**
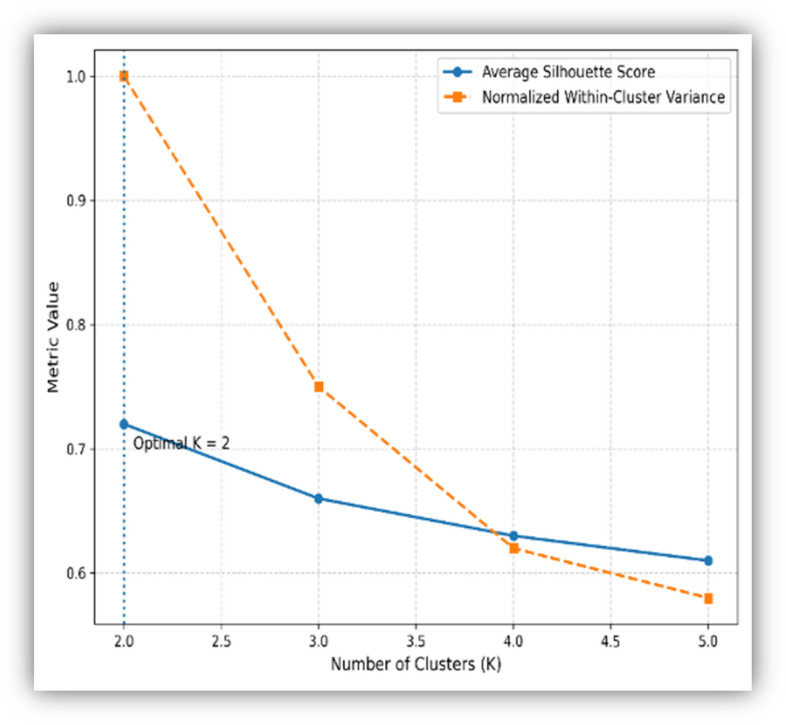
Elbow and Silhouette analysis for the k-means clustering of upward ramp events (average results for 2015–2024).

**Fig. 13:**
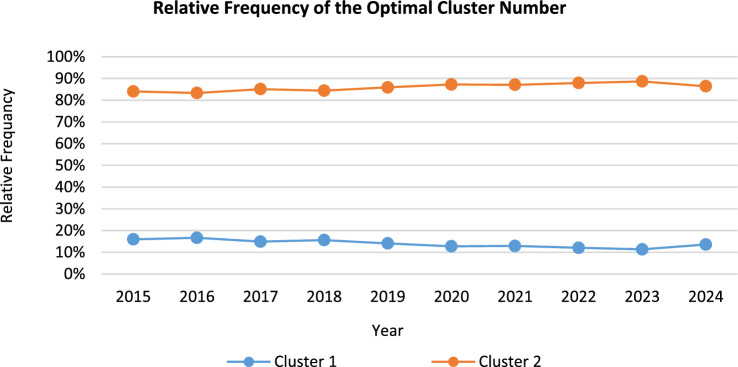
Relative frequency of each cluster in the optimal two-cluster solution for upward ramps over 10 years.

Optimal number of clusters: The optimal number of clusters is evaluated, considering a range from 2 to 5 clusters, using the k-means clustering approach with the Silhouette score and the Elbow method. As shown in Fig. 11, the Silhouette scores over the ten years indicate that two clusters represent the optimal solution. Figure 12 presents a combined Elbow and Silhouette analysis, averaged across 2015–2024. The Elbow method also shows a sharp drop in variance from k = 1 to 2, followed by a gradual flattening beyond k = 2. This pattern corresponds to a distinct “elbow” at k = 2, confirming the same optimal cluster number derived from the Silhouette analysis. Table 6 summarizes the characteristics of these clusters, where cluster 1 corresponds to high power ramps, their relative frequency ranging from 11.3 to 16.7% over the ten years, and cluster 2 corresponds to low power ramps, ranging from 83.3–88.7%. Figure 13 further illustrates that these percentages remain nearly constant over the ten years studied, confirming the stability of the ramp classification.

**Table 6:** Optimal cluster characteristics for upward ramps by year.

Cluster data		Year									
		2015	2016	2017	2018	2019	2020	2021	2022	2023	2024
Cluster 1	Centroid (MW)	61.775	58.848	76.424	85.849	122.664	145.164	168.459	190.658	206.973	177.834
	N of ramps	2759	2909	2578	2707	2450	2224	2235	2103	1956	2343
	Within-cluster variance	866.2	675.03	1313.67	1887.71	3653.36	4545.37	6898.89	8743.2	16,221.14	12,046.57
	Silhouette scores	0.438	0.452	0.432	0.429	0.438	0.438	0.434	0.438	0.422	0.435
Cluster 2	Centroid (MW)	12.775	11.748	14.841	17.166	23.401	26.238	29.069	30.932	36.184	31.533
	N of ramps	14,521	14,542	14,696	14,634	14,937	15,212	15,082	15,323	15,291	14,900
	Within-cluster variance	99.287	88.930	143.551	184.733	361.048	487.282	632.601	758.416	964.725	743.939
	Silhouette scores	0.744	0.748	0.756	0.752	0.761	0.770	0.778	0.789	0.775	0.769

#### Four cluster classification for downward ramps by k-means

**Fig. 14:**
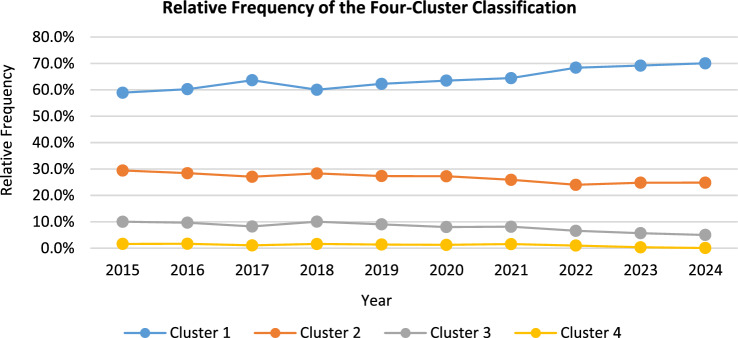
Relative frequency of each cluster in the four cluster classifications.

Table 7 presents the classification results and summarizes the characteristics of each cluster over the ten years. Figure 14 shows that the 10 years have nearly a fixed percentage for each ramp category, as the relative frequency of low power ramps (cluster 1) ranges from 58.9% to 70.1%, medium power ramps (cluster 2) ranges from 24% to 29.4%, high power ramps (cluster 3) ranges from 5 to 10%, and severe power ramps (cluster 4) ranges from 0.1% to 1.7% of the total power ramps.

**Table 7:** Four-cluster classification for downward ramps by k-means.

Cluster data		Year									
		2015	2016	2017	2018	2019	2020	2021	2022	2023	2024
Cluster 1	Centroid (MW)	− 7.08	− 6.77	− 8.60	− 9.59	− 13.41	− 14.60	− 16.18	− 18.44	− 21.77	− 20.31
	N of ramps	10,456	10,648	11,290	10,620	10,987	11,229	11,413	12,042	12,306	12,531
	Within-cluster variance	23.60	22.45	38.47	44.54	90.01	113.98	140.27	199.17	266.85	248.46
	Silhouette scores	0.688	0.693	0.701	0.693	0.69	0.708	0.709	0.716	0.715	0.721
Cluster 2	Centroid (MW)	− 27.12	− 26.63	− 35.20	− 37.61	− 52.85	− 61.43	− 68.37	− 82.00	− 94.95	− 92.38
	N of ramps	5222	5020	4802	5013	4822	4818	4589	4229	4413	4442
	Within-cluster variance	50.08	50.96	90.86	99.02	199.78	288.46	356.63	570.62	766.48	741.16
	Silhouette scores	0.512	0.505	0.51	0.508	0.508	0.505	0.506	0.499	0.501	0.507
Cluster 3	Centroid (MW)	− 58.44	− 58.29	− 78.21	− 81.37	− 115.94	− 138.21	− 153.98	− 195.33	− 228.78	− 227.53
	N of ramps	1779	1708	1459	1778	1593	1416	1439	1161	1010	898
	Within-cluster variance	159.26	165.68	311.90	292.50	657.51	953.30	1231.92	2076.49	3774.57	5076.28
	Silhouette scores	0.493	0.485	0.496	0.506	0.495	0.498	0.483	0.493	0.482	0.458
Cluster 4	Centroid (MW)	− 125.15	− 126.28	− 176.28	− 174.57	− 258.93	− 302.88	− 338.71	− 441.59	− 682.92	− 1175.04
	N of ramps	287	296	190	283	244	221	274	175	59	15
	Within-cluster variance	1209.75	1159.30	3570.81	2847.64	5868.40	7928.11	10,947.26	17,638.54	138,905.20	146,476.54
	Silhouette scores	0.457	0.472	0.452	0.422	0.443	0.436	0.46	0.456	0.342	0.527

**Fig. 15:**
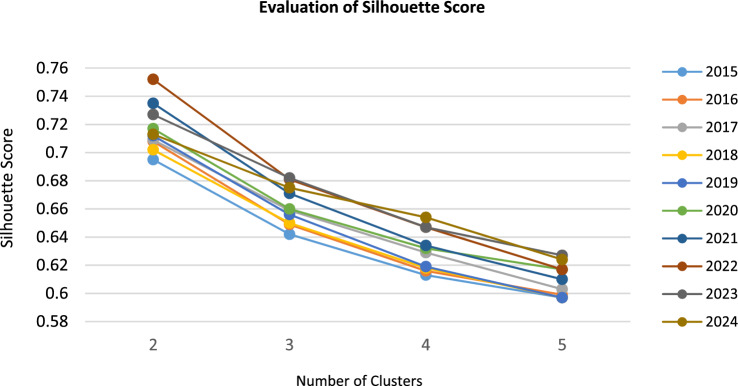
Silhouette score-based evaluation of optimal cluster numbers by year for downward ramps.

**Fig. 16:**
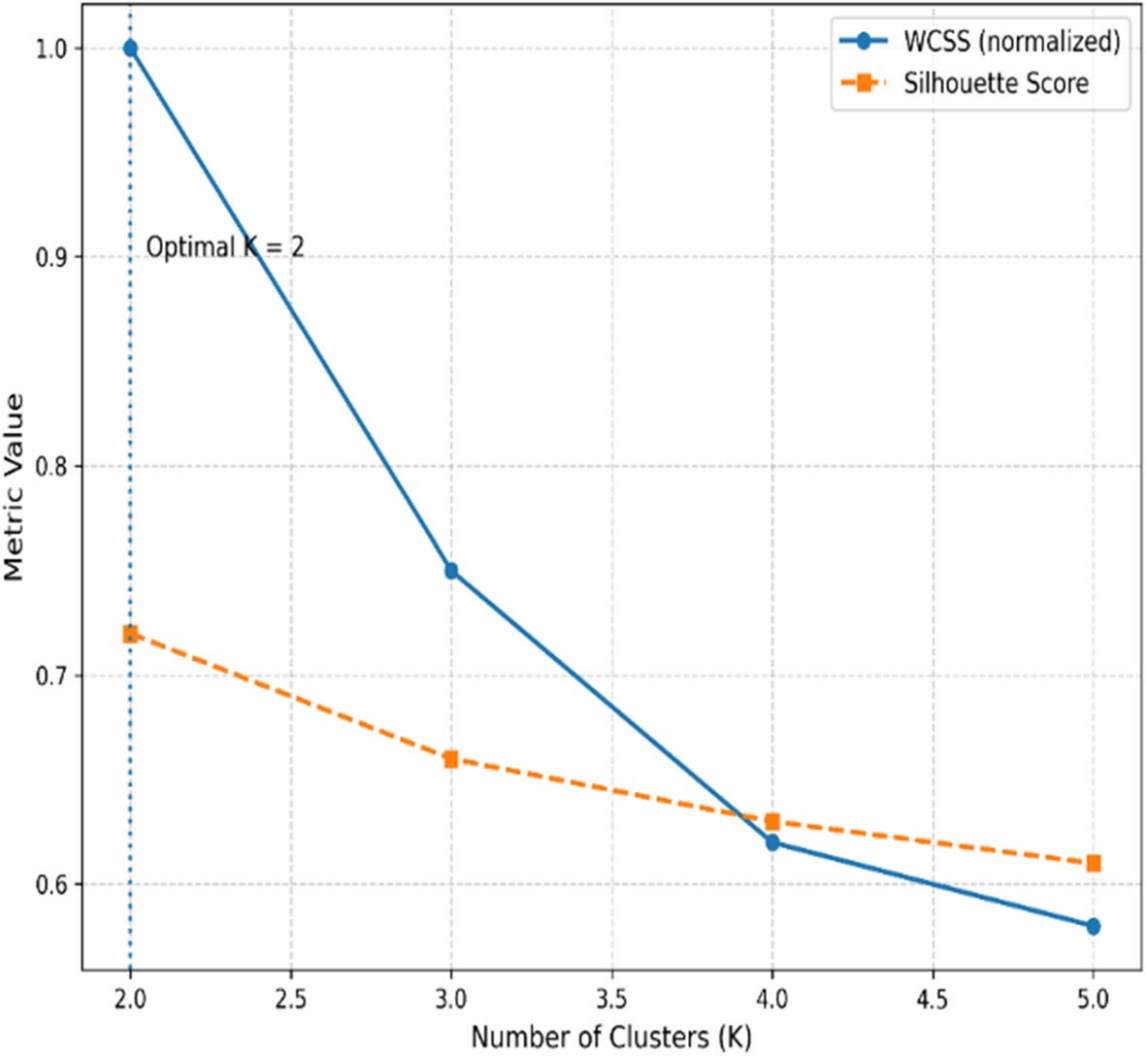
The Elbow and Silhouette analysis for k-means clustering of downward ramp events (2015–2024).

**Fig. 17:**
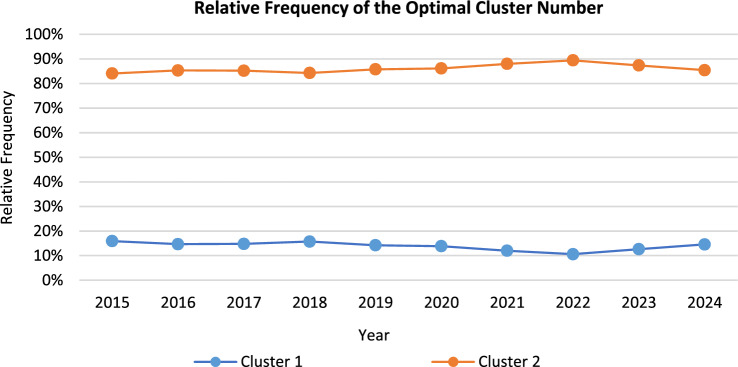
Relative frequency of optimal cluster number.

Optimal number of clusters: The optimal number of clusters, ranging from 2 to 5, is evaluated using the k-means clustering approach with the Silhouette score and the Elbow method. As shown in Fig. 15, the Silhouette scores across the ten years indicate that two clusters represent the optimal solution. Figure 16 shows the Elbow and Silhouette analysis, averaged across 2015–2024. The Elbow method also shows a sharp drop in variance from k = 1 to 2, followed by a gradual flattening beyond k = 2. This pattern corresponds to a distinct “elbow” at k = 2, confirming the same optimal cluster number derived from the Silhouette analysis. Table 8 summarizes the characteristics of these clusters, where cluster 1 corresponds to high power ramps, their relative frequency ranging from 10.6% to 15.9% over the ten years, and cluster 2 corresponds to low power ramps, ranging from 84.1%–89.4%. Figure 17 further illustrates that these percentages remain nearly constant over the ten years studied, confirming the stability of the ramp classification.

**Table 8:** Optimal cluster characteristics for downward ramps by year.

Cluster data		Year									
		2015	2016	2017	2018	2019	2020	2021	2022	2023	2024
Cluster 1	Centroid (MW)	− 60.071	− 61.792	− 74.867	− 83.936	− 119.501	− 137.061	− 168.256	− 198.747	− 190.708	− 164.408
	N of ramps	2823	2592	2622	2781	2510	2448	2125	1863	2249	2605
	Within-cluster variance	772.69	837.46	1415.61	1539.94	3363.83	4495.31	6911.62	10,017.18	14,105.61	11,168.15
	Silhouette scores	0.433	0.429	0.430	0.444	0.431	0.427	0.425	0.425	0.421	0.420
Cluster 2	Centroid (MW)	− 12.464	− 12.102	− 14.377	− 16.837	− 23.131	− 25.401	− 29.203	− 31.922	− 34.066	− 30.007
	N of ramps	14,921	15,080	15,119	14,913	15,136	15,236	15,590	15,744	15,539	15,281
	Within-cluster variance	93.001	93.824	137.578	178.074	347.452	448.973	632.030	822.851	835.798	656.478
	Silhouette scores	0.745	0.756	0.757	0.750	0.758	0.764	0.777	0.790	0.771	0.762

## Zk-means classification results

### Four-category classification based on Zk-means approach for upward and downward ramps

The summary statistics of the first step, by transforming the data into Z-scores, are presented in Table 9, and then the k-means clustering approach is applied. The classification results are comparable to those obtained using the traditional k-means in terms of the relative frequency of each cluster, but they differ in the centroid locations and the within-cluster variance. Table 10 summarizes the centroids and within-cluster variances for each cluster over the ten years as determined by the ZK-means method.

**Table 9:** Summary statistics of Z-score standardization for both types of ramps.

Year	Ramp type	Observations	Minimum (MW)	Maximum (MW)	Mean (MW)	σ (MW)
2015	Upward	17,280	− 0.88	14.60	0	1
	Downward	17,744	− 0.89	12.49	0	1
2016	Upward	17,451	− 0.88	11.09	0	1
	Downward	17,672	− 0.86	13.28	0	1
2017	Upward	17,274	− 0.85	14.85	0	1
	Downward	17,741	− 0.83	22.51	0	1
2018	Upward	17,341	− 0.85	21.66	0	1
	Downward	17,694	− 0.87	13.63	0	1
2019	Upward	17,387	− 0.83	13.66	0	1
	Downward	17,646	− 0.84	14.99	0	1
2020	Upward	17,436	− 0.82	11.30	0	1
	Downward	17,684	− 0.82	13.95	0	1
2021	Upward	17,317	− 0.78	13.83	0	1
	Downward	17,715	− 0.78	16.96	0	1
2022	Upward	17,426	− 0.75	18.35	0	1
	Downward	17,607	− 0.74	16.91	0	1
2023	Upward	17,247	− 0.74	32.87	0	1
	Downward	17,788	− 0.75	34.27	0	1
2024	Upward	17,243	− 0.74	31.76	0	1
	Downward	17,886	− 0.74	32.78	0	1

**Table 10:** Four cluster classification results for both types of ramps by Zk-means.

Cluster data			Year									
			2015	2016	2017	2018	2019	2020	2021	2022	2023	2024
Cluster 1	Upward	Centroid (MW)	− 0.56	− 0.59	− 0.55	− 0.55	− 0.53	− 0.54	− 0.49	− 0.49	− 0.45	− 0.44
		Within-cluster variance	0.05	0.04	0.05	0.04	0.05	0.04	0.05	0.04	0.05	0.06
	Downward	Centroid (MW)	− 0.58	− 0.56	− 0.52	− 0.57	− 0.54	− 0.53	− 0.51	− 0.47	− 0.44	− 0.44
		Within-cluster variance	0.05	0.04	0.05	0.05	0.05	0.05	0.04	0.04	0.05	0.06
Cluster 2	Upward	Centroid (MW)	0.39	0.29	0.37	0.34	0.41	0.32	0.46	0.38	0.48	0.64
		Within-cluster variance	0.12	0.09	0.11	0.10	0.12	0.10	0.13	0.10	0.12	0.17
	Downward	Centroid (MW)	0.32	0.32	0.42	0.33	0.37	0.41	0.38	0.49	0.57	0.64
		Within-cluster variance	0.10	0.10	0.12	0.10	0.10	0.12	0.10	0.13	0.15	0.17
Cluster 3	Upward	Centroid (MW)	1.92	1.59	1.82	1.73	1.97	1.75	2.07	1.84	2.13	2.65
		Within-cluster variance	0.39	0.26	0.35	0.31	0.40	0.32	0.42	0.33	0.51	1.08
	Downward	Centroid (MW)	1.71	1.72	1.96	1.72	1.81	1.95	1.85	2.19	2.42	2.67
		Within-cluster variance	0.32	0.32	0.40	0.30	0.34	0.38	0.36	0.47	0.72	1.14
Cluster 4	Upward	Centroid (MW)	5.43	4.20	4.83	4.82	5.60	4.74	5.57	4.89	6.62	15.48
		Within-cluster variance	3.70	1.84	2.82	4.49	4.01	2.26	3.57	3.35	16.96	34.91
	Downward	Centroid (MW)	4.68	4.72	5.45	4.68	5.08	5.24	5.00	5.89	8.70	16.90
		Within-cluster variance	2.40	2.26	4.54	2.88	3.07	3.18	3.20	3.98	26.59	33.03

The optimal number of clusters, ranging from 2 to 5, evaluated by the Silhouette score and the Elbow method, is the same as the k-means clustering approach.

### Evaluation of the ZK-means approach against its constituent methods

This analysis provides a detailed evaluation of various clustering approaches applied to wind power output data. The discussion incorporates specific clarifications regarding the methodology, interpretations and data analysis. Each method divides power ramps into non-overlapping clusters. The relative frequency of each cluster is nearly constant over the 10 years by the three methods, and cross-validation confirmed that the obtained cluster distributions were consistent, yielding approximately identical relative frequencies for each cluster. k-means and Zk-means results illustrate that the optimal cluster number is achieved by joining low and medium power ramps in one cluster and high and severe ramps in the other one. The range of the relative frequency for each cluster over the 10 years, based on the Z-score method only, is notably the smallest compared to the other clustering methods, as shown in Table 11. This signifies that Z-score standardization efficiently diminishes the dispersion or variance among clusters. The comparative table distinctly demonstrates a notable improvement in cluster homogeneity when utilizing the Z-score for clustering, as opposed to conventional k-means and the suggested Zk-means clustering technique. The progressively narrower “Min” and “Max” ranges for clusters produced by the Z-score method indicate enhanced internal consistency and compactness within these clusters. Data points within clusters delineated by Z-score ranges exhibit greater intrinsic similarity and are closely grouped, enabling each cluster to accurately represent a distinct pattern of wind power output over the decade.

**Table 11:** Comparison between the three clustering techniques based on the relative frequency of clusters over 10 years.

Upward power ramps					
Cluster	ZK-means (min–max range)	k-means (min–max range)	Z-score (min–max range)	Range reduction by Z-score	% Reduction by Z-score
1	58.8–70.5% (11.7%)	58.8–70.5% (11.7%)	64.6–68.5% (3.9%)	7.8%	66.7%
2	23.9–28.9% (5%)	23.9–28.9% (5%)	20.5–23.6% (3.1%)	1.9%	38%
3	5.4–11.1% (5.7%)	5.4–11.1% (5.7%)	6.2–7.8% (1.6%)	4.1%	71.9%
4	0.1–2.1% (2%)	0.1–2.1% (2%)	3.7–4.6%( 0.9%)	1.1%	55%
Downward power ramps					
1	58.9–70.1% (11.2%)	58.9–70.1% (11.2%)	64.5–68.0% (3.5%)	7.7%	68.8%
2	24.0–29.4% (5.4%)	24.0–29.4% (5.4%)	21.6–23.7% (2.1%)	3.3%	61.1%
3	5.0–10.0% (5.0%)	5.0–10.0% (5.0%)	6.1–7.4% (1.3%)	3.7%	74%
4	0.1–1.7% (1.6%)	0.1–1.7% (1.6%)	3.6–4.4% (0.8%)	0.8%	50%

The effects of Z-scores on the row data and clustering can be summarized as follows:

1. Rescaling: It transforms data to have a mean of 0 and a standard deviation of 1, effectively rescales all features to a common range, and eliminates the impact of differing units or magnitudes.
2. Reduced influence of outliers: Centring and scaling the data mitigates the disproportionate impact of extreme values (outliers) on distance estimates, resulting in more compact clusters.
3. Improved distance metrics: Clustering methodologies, such as k-means, often rely on distance metrics (e.g., Euclidean distance). When characteristics display markedly different scales, the feature with the largest range may dominate the distance calculation, irrespective of its significance in assessing similarity. Standardization ensures that all attributes contribute uniformly to the distance calculation.
4. More compact clusters: The normalization of data points allows the algorithm to recognize stronger clustered groups, resulting in a narrower range within each cluster.

The conventional k-means clustering method presents the following challenges:

1. Sensitivity to scale: k-means exhibits considerable sensitivity to the scale of the features. If features have differing ranges, the algorithm will inherently attribute more importance to those with larger ranges, leading to clusters predominantly influenced by those features.
2. Larger cluster ranges: This sensitivity may result in less compact clusters, as the algorithm struggles to accurately group data points when certain dimensions display significantly greater dispersion than others. Outliers can distort cluster centroids, thereby broadening the entire range of the cluster.
3. Suboptimal clustering: Without standardization, distance computations may inadequately reflect the true similarity among data points, leading to suboptimal or less meaningful clusters.

The discovery that the standard score method yields a more restricted range for clusters over a decade than k-means (lacking prior standardization) highlights a significant advantage of data preparation. Standardization improves k-means by producing more stable, interpretable, and compact clusters, ensuring equal contribution from all attributes to the clustering process, rather than permitting dominance by features with larger numerical ranges. Although the frequency range of each cluster over the decade is consistent for both the conventional k-means and the proposed Zk-means clustering method, the amalgamation of Z-score standardization with k-means clustering provides significant benefits concerning the quality, robustness, and interpretability of the clustering process and its resultant cluster attributes, as outlined below:

1. Correct handling of feature scaling discrepancies: The k-means algorithm predominantly depends on distance measurements, typically Euclidean distance, to measure similarity between data points and cluster centroids. In datasets with variables of differing scales or magnitudes (e.g., one feature ranging from 0 to 100,000 and another from 0 to 10), the feature with the larger numerical range can disproportionately influence distance calculations. This leads k-means to disproportionately prioritize numerically dominant features, potentially obscuring more subtle yet significant correlations among data points across varying scales. Consequently, clusters might be formed based on arbitrary magnitude differences rather than true underlying multidimensional resemblances. Z-score standardization transforms data such that each feature is rescaled to possess a mean of zero and a standard deviation of one. This normalization guarantees that all features operate on a comparable scale, thereby contributing equitably to the distance calculations. By eliminating the inherent bias stemming from differing feature scales, Z-score standardization empowers k-means to identify clusters that genuinely reflect the intricate, multidimensional similarity among data points, rather than being tilted by features with exaggerated numerical ranges.
2. Improved cluster quality and cohesion: Even when the volumetric distribution of data points across clusters (i.e., relative frequency) remains quantitatively consistent, the comparison of within-cluster variance for each cluster in Tables 4 and 6 with that in Table 9 shows that applying Z-score standardization preceding k-means leads to clusters with superior internal consistency and compactness (High Intra-cluster Homogeneity). This implies that data points within each cluster are more profoundly similar to one another and are situated in closer proximity to their respective cluster centroids. This elevated internal coherence, in turn, fosters greater inter-cluster separability. When individual clusters demonstrate increased internal uniformity, they consequently become more distinctive and identifiable from other clusters. This enables a more stable and analytically sound division of the data, even though the overall sizes of the resulting groups are numerically similar.
3. Efficient administration of extensive data over prolonged periods: The analysis of extensive datasets accumulated over prolonged periods (e.g., a decade of wind power data) typically reveals the emergence of outliers or significant alterations in data distribution over time. Z-score standardization significantly mitigates the influence of outliers on the clustering process. Assessing data points based on their deviation from the mean within a standardized framework strengthens the robustness and reliability of the clustering process in identifying stable patterns, particularly amid temporal fluctuations or anomalies.
4. Improved interpretability: When clusters are delineated by genuine, multidimensional similarity, resulting from effective data standardization, the analysis of each cluster’s unique attributes becomes more precise and enlightening. Analysts can clearly identify the unique patterns and traits represented by each cluster with greater accuracy and precision. The standardized scale improves understanding of a cluster’s position relative to the overall data average, thereby enhancing the actionable insights derived from the clustering outcomes.

### Comparative analysis of the proposed ZK-means and DBSCAN clustering approaches

#### DBSCAN clustering methodology

DBSCAN proficiently identifies clusters of various shapes and sizes while differentiating noise. In contrast to range-based or centroid-based methods, DBSCAN categorises data points according to their density instead of a predetermined number of clusters. DBSCAN operates by identifying regions of high data point density that are separated by areas of low density, allowing it to form clusters of arbitrary shape while detecting noise points. The algorithm begins by selecting an unvisited point and retrieving all points within its ε-neighborhood. If the number of neighboring points meets or exceeds the threshold MinPts, the point is considered a core point, and a new cluster is formed. The process continues recursively for all directly and indirectly density-reachable points until all points are visited. It necessitates two essential parameters (ε, MinPts), where ε denotes the neighbourhood radius, and MinPts signifies the minimum number of points required to establish a dense region.

#### DBSCAN advantages and disadvantages

Advantages: DBSCAN autonomously identifies the number of clusters, adeptly manages noise, and detects clusters of irregular shapes. It is resilient to outliers and does not impose assumptions regarding the shape of clusters.

Disadvantages: It requires careful tuning of *ε* and *MinPts*, which can vary for different datasets, and struggles when clusters have significantly different densities or when applied to high-dimensional data. It may also produce variable results depending on point ordering.

#### Applications of DBSCAN clustering

DBSCAN has been widely used for data cleaning ^94^, power consumption patterns, anomaly detection^19,99–101^, wind and PV power prediction, and load forecasting^95,96^. For example, in wind energy systems, it has been used to group large turbine clusters into representative sets, boosting both power prediction accuracy and efficiency^97^. Moreover, for microgrid and distributed generation applications, DBSCAN has been combined with deep learning architectures to enhance load forecasting in systems with significant solar output, capturing complex temporal patterns in consumption and generation data^98^. Additionally, electricity theft detection in distribution feeders has been tackled using DBSCAN on spatiotemporal electrical measurements^102^. However, to the best of our knowledge, DBSCAN has not yet been applied to the specific problem of ramp event characterization in wind or PV generation. This paper makes a novel contribution by introducing DBSCAN into this domain, demonstrating its strengths and limitations when used alongside the proposed ZK-means method for clustering ramp events.

#### Results of DBSCAN clustering

Taking *ε* = 10, and the *MinPts* to form a dense region ranging from 2 to 10, Table 12 summarizes the output range of cluster numbers for both types of power ramps over the ten years. The DBSCAN analysis reveals that the range of detected clusters for the upward ramps spans from 1 to 9. Similarly, for downward ramps, the number of clusters ranges from 1 to 8, again showing increasing fragmentation over time. Table 13 illustrates that both upward and downward wind power ramps are characterized by a single, dense cluster that includes over 99% of all ramp observations, indicating a highly cohesive density structure. As the MinPts parameter increases, small clusters are absorbed into the dominant one, while a minor fraction (< 1%) of data points are isolated as noise representing extreme or infrequent ramp events. The increase in noise with higher MinPts was gradual and systematic, confirming the robustness of DBSCAN’s density-based classification in separating typical ramps from rare extremes. Clustering stability is reached for *MinPts* ≥ 6 in upward ramps and *MinPts* ≥ 5 in downward ramps, confirming that downward ramps form slightly denser and more compact distributions. The consistent relative frequencies across all years demonstrate the robustness and temporal stability of DBSCAN in detecting ramp patterns without prior assumptions about cluster count or shape.

**Table 12:** Range of DBSCAN cluster numbers for upward and downward ramps across 2015–2024.

Year	Range of clusters (*ε* = 10, *MinPts* = *2 → 10)*	
	Upward ramps	Downward ramps
2015	1 → 5	1 → 4
2016	1 → 2	1 → 2
2017	2 → 3	1 → 4
2018	2 → 3	2 → 4
2019	1 → 5	1 → 4
2020	1 → 5	1 → 6
2021	2 → 8	1 → 5
2022	2 → 9	1 → 8
2023	2 → 4	2 → 8
2024	1 → 7	2 → 8

**Table 13:** DBSCAN clustering behavior for upward and downward ramps (2015–2024).

Aspect	Upward ramps	Downward ramps	Interpretation
Onset of stability	MinPts ≥ 6	MinPts ≥ 5	Downward ramps stabilize earlier
Main cluster frequency	~ 99.2%	~ 99.6%	Downward ramps are denser
Noise growth with MinPts	Moderate → 0.5%	Slightly lower (≤ 0.4%)	Fewer outliers in downward data
Temporal pattern (2015–2024)	Stable single-cluster structure	Stable single-cluster structure	Consistent density structure across years

These results validate DBSCAN’s suitability for ramp classification, effectively distinguishing the main operational ramp regimes from atypical outliers based solely on data density. Consequently, although DBSCAN excels at identifying complex spatial structures and isolating noise points, its performance is highly sensitive to the choice of *ε* and *MinPts*. Without careful parameter tuning, it often produces highly variable cluster counts, in contrast to the more stable and interpretable clustering patterns achieved by the other methods.

### Computational comparison of the four clustering approaches: Z-score, k-means, DBSCAN and ZK-means

The following discussion evaluates the computational performance of these methodologies. The computational time is directly related to energy consumption. The results demonstrate that, although the proposed ZK-means method exhibits a slight increase in computational time compared to its constituent methods, it remains faster than DBSCAN. Furthermore, the enhanced interpretability and operational insights provided by the proposed approach offer critical advantages for efficient grid management, justifying this minor trade-off. The computational complexity has been assumed for each algorithm according to the theoretical calculation steps. The computational complexity of Z-score clustering is expressed by Eq. (9) as follows:

9 $$O\left( {n*d} \right)$$

where *n* is the number of data points, *d* is the number of dimensions, and the big *O* notation provides an upper bound on the growth rate of the computational time. Each data point’s features are processed once for the computation of the mean and standard deviation, and then for transformation and comparison against predefined range thresholds without iterative optimization loops. This method is computationally lightweight, highly scalable, and theoretically the fastest among the three. The energy consumption is low, making it well-suited for very large datasets. This linear complexity arises because only a single pass over the dataset is needed for standardization and cluster assignment. The computational complexity of k-means clustering is expressed by Eq. (10) as follows:

10 $${\mathrm{O}}({\mathrm{k}}*{\mathrm{n}}*{\mathrm{d}}*{\mathrm{i}})$$

where *k* is the number of clusters, and *i* is the number of iterations. Each iteration typically involves calculating the distance between *n* data points and *k* centroids across *d* dimensions, and the number of iterations *i* is generally small and either fixed or determined by a convergence criterion. The speed/time is considered computationally efficient and relatively fast for large datasets where *k* and *d* are not excessively large. The energy consumption is considered moderate. The computational complexity of the DBSCAN clustering method is expressed by Eq. (11) as follows:

11 $$0(n\log n)$$

When spatial indexing structures, such as KD-trees or R*-trees are used, the computational complexity is expressed by Eq. (12) as follows:

12 $$0(n^{{2}} )$$

DBSCAN is generally slower than k-means for very large or high-dimensional datasets due to repeated neighborhood queries, but it provides superior robustness in detecting non-spherical clusters and outliers. The energy consumption is typically higher than that of k-means, as it scales with the number of distance computations performed during density estimation and neighborhood expansion. The computational complexity of the proposed Zk-means clustering approach is the sum of its components, thus can be expressed as O(n*d) + O(k*n*d*i), which simplifies to O(k*n*d*i) since normalization adds only a linear overhead. In terms of energy consumption, the method remains moderate, though slightly higher than k-means alone due to the additional preprocessing stage.

As illustrated in Table 14, the four clustering approaches exhibit distinct trade-offs among computational speed, accuracy, and scalability. The Z-score method is the most efficient and energy-conserving, making it ideal for rapid partitioning of large datasets, though it may sacrifice clustering precision in highly variable data. The conventional k-means algorithm optimizes the balance between efficiency and accuracy, offering adaptable clustering capabilities but with increasing computational demands in high-dimensional spaces. The proposed hybrid ZK-means technique further enhances this balance by integrating the stability of normalization with the scalability of k-means, providing a robust and pragmatic solution for large-scale, high-dimensional clustering tasks. In contrast, DBSCAN excels in detecting irregular, non-spherical clusters and identifying outliers, but its sensitivity to parameter selection (ε and MinPts) and reduced scalability limit its suitability for consistent, long-term ramp characterizations.

**Table 14:** Cross-method comparison between range-based, centroid-based, and density-based clustering approaches.

Criterion	DBSCAN (density-based)	k-means (centroid-based)	ZK-means (centroid-based)	Z-score (range-based)
Clustering principle	Density-based; regions of high point density separated by sparse areas	Centroid-based; minimizes within-cluster variance	Hybrid: Z-score normalization + centroid update	Range-based thresholding over standardized values
Requires predefined k?	No	Yes	Yes	Yes—Predefined by range thresholds
Parameter sensitivity	High (ε, MinPts)	Moderate (k)	Moderate	Low (predefined thresholds)
Noise/Outlier handling	Explicit (noise labeled separately)	Weak (absorbed into clusters)	Improved (normalization mitigates outliers)	Strong (excluded via predefined thresholds)
Cluster shape	Arbitrary, non-spherical	Spherical/elliptical	Compact, statistically consistent	Range-limited, non-parametric
Temporal stability	Low; highly variable across years	Moderate	High; stable across time and scales	Very high; fixed by statistical thresholds
Relative frequency of clusters	Few dense clusters + sparse noise (< 10%)	Uniform sizes	Adaptive to data variability; preserves true distribution	Fixed by selected Z thresholds
Interpretability	Moderate; requires density visualization	High	High; combines interpretability and adaptive scaling	Very high; intuitive threshold-based interpretation
Main strength	Detects irregular/rare ramp patterns without prior k	Simple, fast, consistent global partitioning	Balances adaptability and interpretability; improved detection of rare/transition ramps	Intuitive, stable, scale-independent representation
Main limitation	Sensitive to ε and MinPts; unstable cluster counts	Overlooks extreme or rare events	Slightly higher computational cost than k-means	Limited adaptability to evolving distributions
Computational complexity	O(n log n) with spatial indexing (otherwise O(n^2^))	O(k*n*d*i)	≈ O(k*n*d*i); normalization adds linear overhead	O(n*d) (single pass)
Computational speed	Moderate to slow (density queries)	Fast (iterative but efficient)	Moderate (faster convergence via normalization)	Fastest (direct assignment)
Scalability	Moderate; depends on density and parameters	Good for moderate k and d	Enhanced by normalization; robust for large-scale data	Excellent; linear scaling
Suitability for long-term trend analysis	Weak	Good	Excellent (balances precision and temporal stability)	Best (consistent benchmark reference)
Common validation metrics	Silhouette, ARI, Davies–Bouldin	Silhouette, ARI	Silhouette, ARI	Silhouette (range-based validity)
Overall role/advantage	Detects arbitrary-shaped clusters and anomalies; useful for exploratory density analysis	Widely used baseline for global partitioning	Hybrid improvement combining Z-score normalization with k-means for robust, interpretable ramp characterization	Benchmark range-based method; fast, stable, and interpretable for operational classification

The Z-score (Range-Based) method offers flexibility in adjusting the number of clusters (*k*) and defining noise levels, as both can be predetermined through the selection of Z-score interval ranges. By modifying these ranges, the analyst can easily control the clustering granularity and the threshold for identifying outliers or noise before classification.

## Limitations and future work

Although the proposed ZK-means technique demonstrated effective performance in identifying and classifying wind power variations, several limitations should be acknowledged. The case study was conducted using aggregated national-level wind generation data from Belgium, which may smooth out local variability and extreme ramp events. Applying the method to higher-resolution or plant-level datasets could reveal more detailed ramp dynamics and enhance the robustness of the classification outcomes.

In addition, the quality, completeness, and spatial representativeness of the available wind generation data directly influence the clustering results. Because the outputs of the ZK-means classification are spatially dependent, applying the method to a different location or dataset may yield variations in the relative frequency and distribution of clusters. Hence, the model’s transferability should be interpreted cautiously, and local calibration may be required.

From a methodological perspective, the silhouette coefficient initially indicated two optimal clusters corresponding to low and high ramp events. To improve discrimination among ramp magnitudes and enhance operational interpretability and control. Also, to avoid the drawbacks of binary classification, this configuration was refined to four clusters. This refinement provided a more granular representation of ramp behavior without compromising consistency or stability across years.

Furthermore, the current implementation relied solely on power time-series features, without incorporating meteorological variables such as wind speed, direction, or pressure gradients. Integrating such multi-source information could strengthen the physical interpretability of the derived clusters and improve their linkage to underlying weather patterns. Combining meteorological and generation-based features would also allow the method to distinguish between structurally different ramp causes (e.g., weather-driven vs. grid-induced). Although the method achieved reasonable computational efficiency, its scalability to large spatio-temporal datasets and real-time operation still requires further evaluation. Implementing parallel processing or streaming-based clustering architectures could help address this challenge. The algorithm’s hybrid structure, combining normalization with centroid-based partitioning, makes it promising for adaptation to real-time, incremental clustering frameworks.

For future work, the ZK-means framework can be extended through the integration of unsupervised feature learning methods (e.g., autoencoders or transformers) to extract latent representations of ramp events. It can also be combined with probabilistic forecasting or operational decision-support modules to assist grid balancing and tested across different countries and renewable energy mixes to assess its generalization capability. Finally, incorporating uncertainty quantification into the ZK-means clustering process may provide confidence bounds for ramp classifications, enhancing its practical utility for grid operations.

## Conclusion

The comparative evaluation among the three clustering paradigms, range-based (Z-score), centroid-based (k-means and ZK-means), and density-based (DBSCAN), demonstrates a clear progression from simplicity and interpretability to adaptivity and statistical robustness. The Z-score method provided a transparent and interpretable framework for ramp classification. Its strength lies in defining ramps using statistical boundaries (± Zσ), which enables flexible thresholding without requiring complex tuning parameters. This approach offered a reliable baseline for identifying ramp magnitudes and their relative frequency distribution across years.

The proposed ZK-means algorithm builds directly upon this foundation by integrating Z-score normalization with the centroid-driven structure of k-means, creating a hybrid model that merges statistical consistency with clustering adaptability. This combination allows for automatic balance between normalization and partitioning, enabling the method to:

- Adapt to varying data scales and interannual variability;
- Preserve the statistical distribution of ramp magnitudes across different years; and
- Enhance sensitivity to rare or extreme ramp events that traditional k-means tends to overlook.

Consequently, the ZK-means delivers more representative cluster centroids and lower intra-cluster variance, improving both the stability and interpretability of long-term ramp characterization.

In contrast, DBSCAN, a density-based approach, showed strong performance in identifying irregular and isolated ramp events and explicitly labeling outliers, but its effectiveness was constrained by the sensitivity to *ε* and *MinPts* selection, leading to variable cluster counts, and making it less scalable for yearly wind data with variable densities.

In summary, while each method provides distinct analytical value, DBSCAN for density-driven anomaly detection, k-means for simplicity and speed, and Z-score for clarity and interpretability, the proposed ZK-means method offers the most balanced performance. It combines the statistical rigor of Z-score method with the adaptive clustering capability of k-means, resulting in a generalized, flexible, and robust framework for wind ramp event analysis. This hybridization represents a significant methodological advancement, bridging the gap between range-based interpretability and centroid-based adaptivity, an essential contribution for scalable, multi-year ramp characterizations in renewable energy datasets.

## Data Availability

The datasets analyzed during the current study are available in ^103^ (a publicly accessible source). The data were used in accordance with the terms and conditions of the provider, which permit their use for academic research and the publication of derived results in an open-access journal.
